# U‐Shaped Response of Flowering Time to Low and High Nitrogen via a Transcription Feedback Loop in Rice

**DOI:** 10.1002/advs.202508498

**Published:** 2025-12-07

**Authors:** Shunan Zhang, Guanzhong Shou, Xianping Li, Xuemin Song, Xuesong Li, Junjie Guo, Jiaqiang Lu, Yi Jin, Hongye Qu, Ming Yan, Wona Ding, Ying Liu, Guohua Xu

**Affiliations:** ^1^ National Key Laboratory of Crop Genetics & Germplasm Enhancement and Utilization Nanjing Jiangsu 211800 China; ^2^ Key Laboratory of Plant Nutrition and Fertilization in Low‐Middle Reaches of the Yangtze River Ministry of Agriculture Nanjing Agricultural University Nanjing Jiangsu 211800 China; ^3^ State Key Laboratory of Biocontrol Guangdong Provincial Key Laboratory of Plant Stress Biology School of Agriculture and Biotechnology Shenzhen Campus of Sun Yat‐sen University Sun Yat‐sen University Shenzhen Guangdong 518107 China; ^4^ MOA Key Laboratory of Grain Crop Genetic Resources Evaluation and Utilization Shanghai Agrobiological Gene Center Shanghai 201106 China; ^5^ Ningbo Key Laboratory of Agricultural Germplasm Resources Mining and Environmental Regulation College of Science and Technology Ningbo University Ningbo 315300 China

**Keywords:** flowering time, nitrogen, transcript factor

## Abstract

Nitrogen (N) availability regulates flowering time (heading date) in rice through a U‐shaped response, where both deficient and superior N delay flowering. This N‐dependent plasticity of flowering time impacts productivity, N use efficiency and rotation schedules, while the underlying mechanisms remain unclear. Here, a reciprocal feedback loop between two transcript factors is identified, N‐mediated heading date 1(Nhd1) and Ghd7, that orchestrates this U‐shaped response under long‐day condition. Deficient‐N delays flowering via repressing *Nhd1* regulated by Ghd7, while superior‐N delays flowering by activating *Ghd7* mediated through glutamine‐induced Nhd1. Genetic and molecular evidence further demonstrates that Heading date 3a (Hd3a) is mainly required for the U‐shape response regulated by the Nhd1‐Ghd7 module. Notably, natural variation analysis reveals that antagonistic combinations of *Nhd1* and *Ghd7* alleles are selected during rice domestication and correlate with geographic patterns of soil N deposition. Furthermore, Nhd1 alleles differ in both transcriptional activity and protein function, enabling fine‐tuning of flowering sensitivity to N availability in weak/none alleles of *Ghd7*. Collectively, this study identifies an Nhd1–Ghd7 regulatory module that regulates the U‐shaped flowering response to N, offering mechanistic insight and potential targets for breeding N‐resilient rice.

## Graphical abstract

The “seesaw effect” of Nhd1‐Ghd7 module in regulating flowering time (days to heading in rice) responses to nitrogen (N) via Hd3a/RFT1: deficient‐N (DN) delays flowering via Ghd7‐mediated suppression of *Nhd1*; moderate‐N (MN) impairs this suppression, enhancing Nhd1 to promote flowering; whereas superior‐N (SN) delays flowering by Nhd1‐activated expression of *Ghd7*.

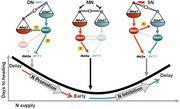

## 1 Introduction

Optimal flowering timing is critical for crop productivity and rotation. Proper flowering ensures sufficient growth duration to maximize the use of light, heat, water, and nutrients.^[^ ^1^, ^2^, ^3^, ^4^, ^5^, ^6^ ^]^ Flowering time is influenced by multiple environmental factors, including temperature, photoperiod (day length), light intensity, water availability, and nutrient status.^[^ ^7^, ^8^, ^9^ ^]^ In rice, the photoperiod‐dependent flowering pathway comprising *OsGIGANTEA (OsGI)*, *Heading date‐1 (Hd1)*, and *Heading date‐3a (Hd3a)* has been well characterized.^[^ ^10^ ^]^ Hd1, unlike its homolog *CONSTANS*, promoting flowering in Arabidopsis,^[^ ^11^ ^]^ has a dual role in rice: accelerating flowering under short‐day (SD) conditions, but delaying it under long‐day (LD) conditions.^[^ ^12^, ^13^ ^]^ Additionally, rice employs *Early heading date‐1 (Ehd1)* to upregulate *Hd3a* and *RFT1*, further accelerating flowering.^[^ ^14^ ^]^

Nitrogen (N) plays a critical role in modulating flowering time. In the field, it is often observed that excessive N delays flowering and maturation.^[^ ^6^, ^15^, ^16^ ^]^ Typically, the response of flowering time to N availability follows a U‐shaped curve, where either deficient‐N (low N) or superior‐N (high N) delays flowering, while moderate‐N promotes flowering. This pattern has been firmly demonstrated in both Arabidopsis^17^ ^]^ and rice (*Oryza sativa*).^[^ ^6^, ^9^ ^]^ Whereas, the thresholds (ranges) for deficient, moderate and superior N levels on the U‐shaped curve vary depending on growth conditions, plant species,^[^ ^6^, ^16^, ^17^ ^]^ as well as two dynamic factors ‐ plant N demand and soil N availability both of which change across growth stages.^[^ ^18^, ^19^, ^20^ ^)^

The delayed flowering under both low and high N may reflect different adaptive strategies in plant. The deficient‐N induced delay represents a survival strategy, which is characterized by postponed growth to save energy until optimal conditions return.^[^ ^21^, ^22^, ^23^ ^]^ In contrast, superior‐N induced delay may enable plants to maximize biomass accumulation via sufficient utilization of resources and energy.^[^ ^24^, ^25^, ^26^ ^]^ These contrasting strategies suggest that the underlying mechanisms of deficient‐N and superior‐N delayed flowering should be distinct.^[^ ^27^ ^]^ In Arabidopsis, several regulators have been characterized in promoting flowering under moderate‐N condition, e.g., the putative nitrate transceptor, NRT1.13, and the BHLH transcription factor, FBH4.^[^ ^27^, ^28^ ^]^ However, how plant induces delayed flowering in response to both deficient‐ and superior‐ N remains largely unknown in crops.^[^ ^9^, ^17^ ^]^

We have previously identified a circadian clock component, Nhd1 (OsCCA1/LHY1), that functions in flowering promoter by directly activating *Hd3a* expression.^[^ ^9^ ^]^ Knockout of *Nhd1* leads to insensitivity of flowering time to N supplies at a broad range.^[^ ^9^ ^]^ Interestingly, under superior‐N, *Nhd1* expression increases, but flowering time is still delays, indicating that additional flowering repressors counteract the effect of Nhd1.^[^ ^6^, ^9^ ^]^ Notably, Ghd7 (Grain size, height and heading date‐7) is known as a flowering repressor under LD condition,^[^ ^29^, ^30^ ^]^ and its expression decreases as N supply increases.^[^ ^31^ ^]^ This observation led us to hypothesize that Ghd7 may function as a flowering inhibitor that interacts with Nhd1 to mediate N‐dependent flowering responses in rice.

In this study, we reveal that Nhd1 and Ghd7 form an N‐responsive feedback loop that mediates the flowering response to N availability in rice. Ghd7 suppresses *Nhd1* expression under low N condition; conversely, Nhd1 upregulates *Ghd7* under high N condition. This reciprocal regulation generates the characteristic U‐shaped flowering response curve to N supplies.

## 2 Results

### 2.1 Nhd1 and Ghd7 Antagonistically Control Flowering Time and Exhibit Distinct Expression Patterns in Response to N

**Figure 1:**
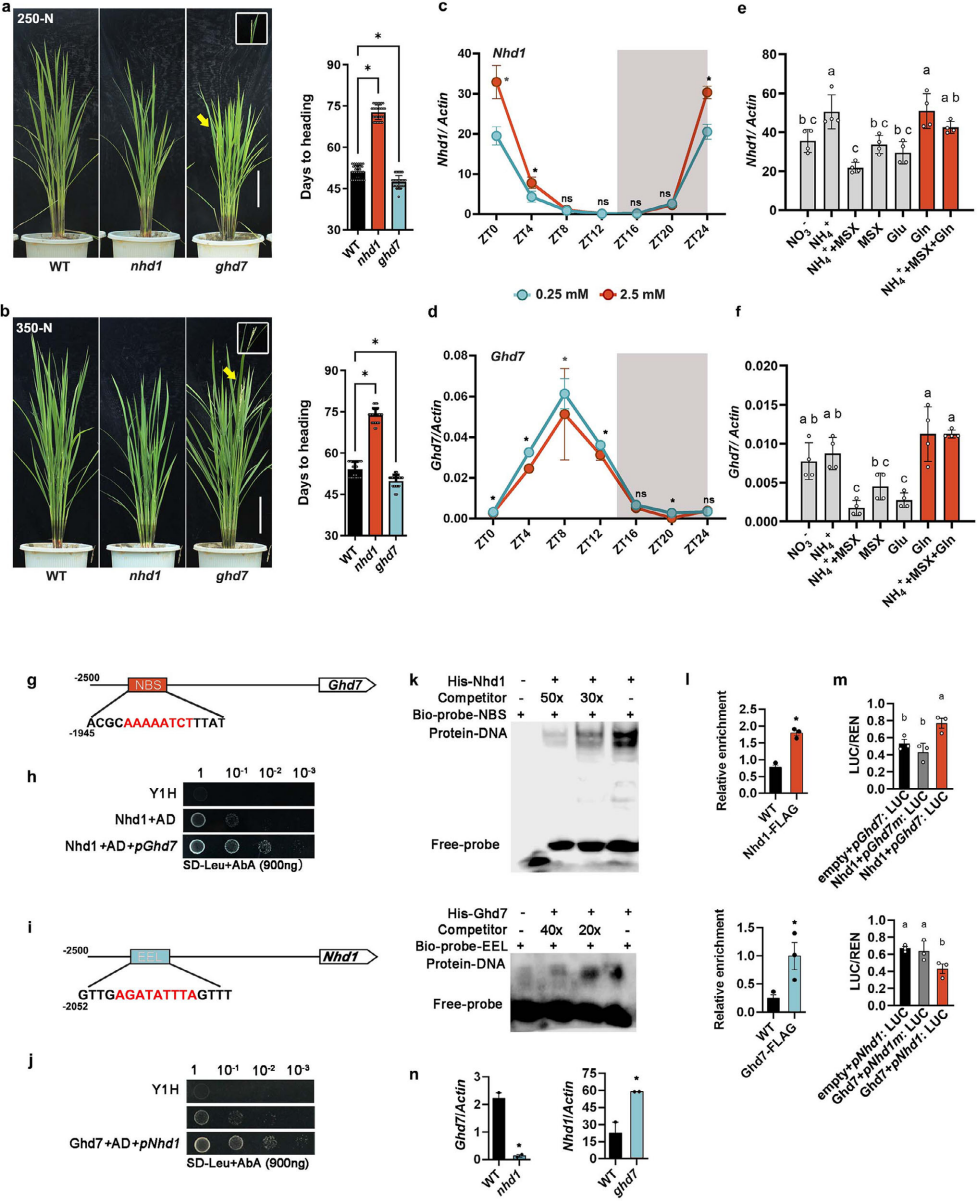
Nhd1 and Ghd7 have opposite effects on flowering regulation and transcriptional response to N supply, forming a positive‐negative transcript loop by binding respective promoter. Flowering phenotype and flowering time (heading date) of WT, *nhd1* and *ghd7* mutants grown in paddy field under a) 250 kg N ha^−1^ and b) 350 kg N ha^−1^ supplies. The arrows and the amplified frames representative spiles in each plant. Scale bar, 20 cm. Student's *t* test was used for the statistical analysis (*p* ≤ 0.05). Diurnal expression pattern of c) *Nhd1* and d) *Ghd7* under 0.25 mm and 2.5 mm N concentrations. The N resource is NH_4_^+^. Plants were grown in hydroponic system and the shoot of each line was sampled for analysis. Values are means±SE (*n* ≥ 3). Student's *t* text is used for the statistical analysis. (*p*≤0.05). e,f) Relative expression of *Nhd1* and *Ghd7* under different N forms. The N concentration in each treatment is 2.5 mm. Values are means±SE (*n* ≥ 3). One‐way ANOVA was used for the statistical analysis (*p*≤0.05). Schematic of NBS (Nhd1 binding site) in g) *Ghd7* promoter and EEL (evening element like motif) in i) *Nhd1* promoter. Binding activity of h) Nhd1 to *Ghd7* promoter and that of j) Ghd7 to *Nhd1* promoter using yeast one‐hybrid system. k) In vitro binding activity of Nhd1 to NBS (upper) and that of Ghd7 to EEL (down) using EMSA. l) ChIP‐qPCR analysis of FLAG‐Nhd1 mediated enrichment of NBS from *Ghd7* (upper) promoter and FLAG‐Ghd7 mediated enrichment of EEL from *Nhd1* promoter (down). m) Transactivation of Nhd1 on the *Ghd7* promoter (upper) and that of Ghd7 on the *Nhd1* promoter (down) in rice protoplast. The sequences with random mutations in each binding site (*pGhd7m* and *pNhd1m*) were used as negative controls. The detail of the mutation sites was shown in material and method “Dual‐luciferase report assay.” One‐way ANOVA was used for the statistical analysis (*p* ≤ 0.05). n) Relative expression of *Ghd7* and *Nhd1* in WT, *nhd1* and *ghd7* mutants. Values are means±SE (*n* ≥ 3). Student's *t* text is used for the statistical analysis (*p* ≤ 0.05).

We hypothesized that Ghd7 may function as a flowering inhibitor that interacts with Nhd1 to mediate superior N‐induced flowering delay. Therefore, we first compared the roles of Nhd1 and Ghd7 in regulating flowering time with supplies of 250  and 350 kg N ha^−1^ at paddy field, which are common superior levels of N.^[^ ^32^, ^33^ ^]^ Consistent with their known phenotypes, *nhd1* mutants exhibited significantly delayed flowering time, while *ghd7 mutants* (Figure S1a, Supporting Information) exhibited earlier flowering time than wild‐type (WT) plants under both N regimes (**Figure** **1**a–d). Moreover, *ghd7* mutants shortened plant height but increased tillering across all growth stages, whereas *nhd1* mutants eventually reached similar height and tiller number as WT at maturity (Figure S1b,c, Supporting Information), indicating that Nhd1 and Ghd7 independently control flowering time and plant architecture. We further analyzed diurnal expression patterns of *Nhd1* and *Ghd7* under different N conditions. The results showed that expression of *Nhd1* peaked in the early daytime and was upregulated by high N (NH_4_^+^) (Figure 1c), while expression of *Ghd7* peaked in afternoon and was downregulated by high N (Figure 1d). We previously found that glutamine (Gln), not NH_4_^+^, activates *Nhd1* transcription (Figure 1e;^[^ ^9^ ^]^). Interestingly, the expression of *Ghd7* was also induced by Gln (Figure 1f). These results indicate that Ghd7 may counteract Nhd1 in mediating N‐dependent flowering responses.

### 2.2 Transcriptional Regulation Between Nhd1 and Ghd7

Nhd1 is known to regulate gene expression by directly binding to a conserved cis‐element (NBS, Nhd1 binding site) in both Arabidopsis and rice.^[^ ^9^, ^34^, ^35^, ^36^ ^]^ Our analysis identified an NBS motif in the *Ghd7* promoter (Figure 1g). Ghd7 was previously shown to bind evening element‐like (EEL) motifs,^[^ ^31^ ^]^ which were found in the *Nhd1* promoter (Figure 1i). Using yeast one‐hybrid (Y1H) and electrophoretic mobility shift assays (EMSA), we confirmed that both Nhd1 and Ghd7 can bind each other's promoter in vitro (Figure 1h,j,k). Chromatin immunoprecipitation (ChIP) assays with *35S*::*Nhd1*::FLAG and *Ubi*::*Ghd7*::FLAG transgenic lines further demonstrated binding activity of Nhd1 to the NBS motif in the *Ghd7* promoter and Ghd7 to the EEL motif in the *Nhd1* promoter in vivo (Figure 1l). Dual‐luciferase reporter (DLR) assays in rice protoplasts revealed that Nhd1 activates *Ghd7* expression, while Ghd7 represses *Nhd1* expression (Figure 1m). Supporting these findings, *nhd1* mutants showed strongly reduced *Ghd7* expression, whereas *ghd7* mutants exhibited enhanced *Nhd1* expression (Figure 1n). Collectively, these results demonstrate that Nhd1 and Ghd7 form a reciprocal transcriptional regulatory loop through direct promoter binding.

### 2.3 Activation of *Ghd7* by Nhd1 is Glutamine‐Dependent, While Suppression of *Nhd1* by Ghd7 is at N‐Deficient Condition

**Figure 2:**
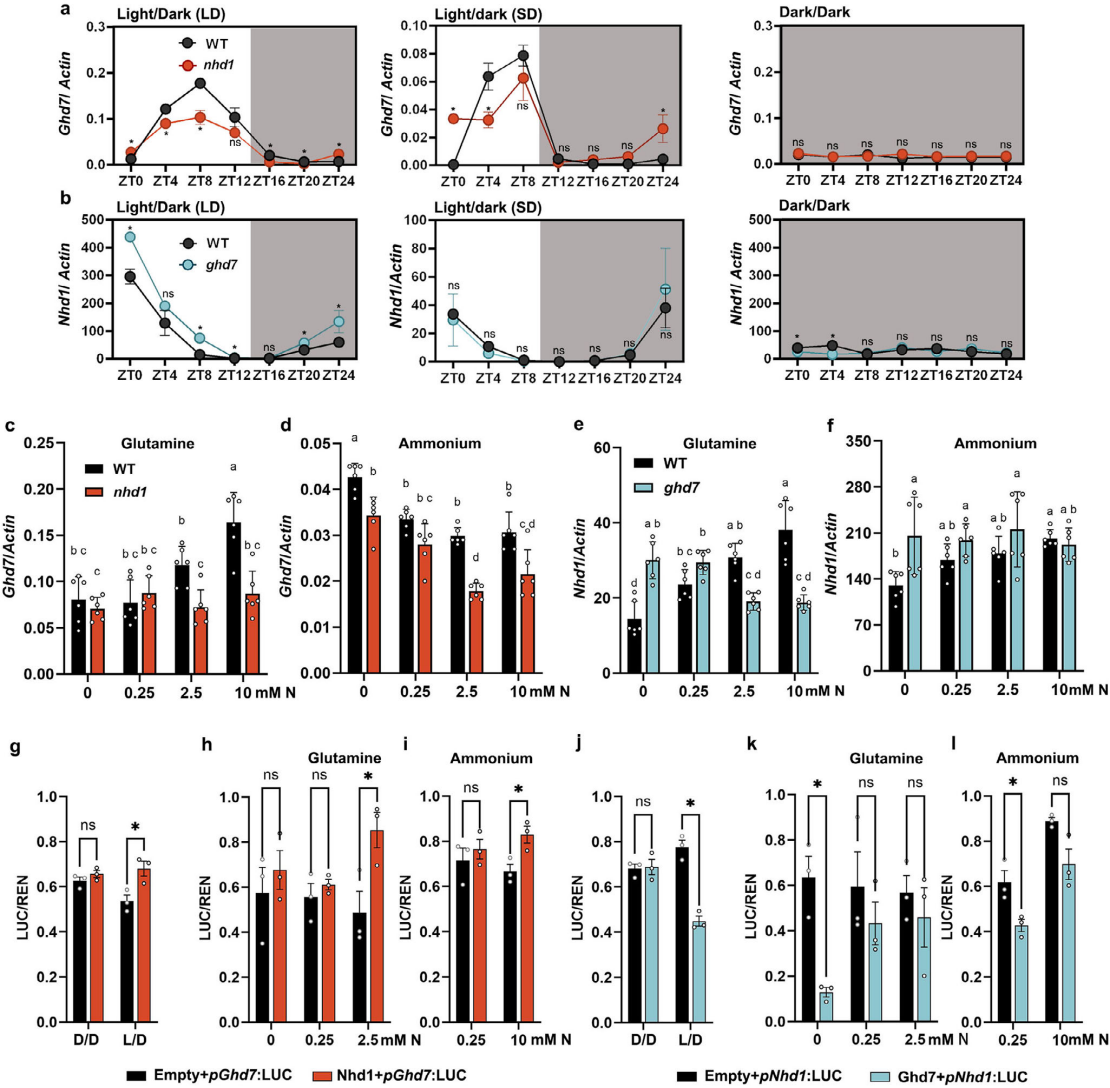
Reciprocal transcriptional regulation of Nhd1 and Ghd7 is affected by change of photoperiod and N supply. Diurnal expression of a) *Ghd7* and b)*Nhd1* in WT, *nhd1* and *ghd7* mutant under long day (LD, light 14 h per dark 10 h), short day (SD, light 10 h per dark 14 h), and constant dark (dark/dark) treatments. Plants were grown in hydroponic system for 40 d and transferred to each treatment for 3 d. The shoot of each line was used for analysis. Values are means±SE (*n* ≥ 5). Student's *t* text is used for the statistical analysis. (*p* ≤ 0.05). Relative expression of *Ghd7* in WT, *nhd1* mutant under different concentrations of c) glutamine and d) ammonium. Relative expression of *Nhd1* in WT and *ghd7* mutant under different concentrations of e) glutamine and f) ammonium. The fully expanded youngest leaf of each plant was sampled at ZT4 for analysis. Transactivation of g–i) *Nhd1* on the *Ghd7* promoter and that of j–l) Ghd7 on the *Nhd1* promoter in rice protoplast under different circadian and N conditions. D/D, dark/dark; L/D, light/dark. Values are means±SE (*n* ≥ 5). One‐way ANOVA was used for the statistical analysis (ns, *p*>0.05; *p ≤ 0.05).

We first investigated the transcriptional interplay between Nhd1 and Ghd7 under different photoperiods. Under rhythmic light conditions (short day, SD; long day, LD), both genes maintained robust diurnal expression patterns in WT, while losing rhythmicity in continuous light (L/L) or dark (D/D) environments (Figure 2a,b; Figure S2, Supporting Information). Notably, loss‐of‐function of *Nhd1* reduced amplitude and advanced phase of *Ghd7* expression in both SD and LD conditions (**Figure** **2**a), whereas loss‐of‐function of *Ghd7* increased *Nhd1* expression under LD condition (Figure 2b), consistent with known photoperiod‐dependent protein degradation of Ghd7 during SD nights.^[^ ^29^, ^30^ ^]^

We further confirmed that the expression of *Ghd7* has a contrast pattern in response to Gln and NH_4_^+^ supplies (Figure 2c,d). Notably, knockout of *Nhd1* decreased expression of *Ghd7* only at high Gln and NH_4_^+^ levels (2.5 mm and 10 mm) (Figure 2c,d). In contrast, knockout of *Ghd7* increased expression of *Nhd1* only at deficient‐N level (0 mm) (Figure 2e,f). Dual‐luciferase reporter assays further elucidated that the interplay between Nhd1 and Ghd7 occurred in LD (Figure 2g,j). The induction of *Ghd7* transcripts by Nhd1 occurred in superior Gln (2.5 mm) or NH_4_^+^ (10 mm) conditions (Figure 2h,i), while Ghd7 suppressed transcripts of *Nhd1* in deficient‐N condition (0 mm) (Figure 2k‐i). These results demonstrated that the induction of *Ghd7* by Gln requires Nhd1, revealing that the superior‐N with a high level of Gln upregulates the expression of *Ghd7* in a Gln‐Nhd1 mediated manner. These comprehensive findings demonstrate that the Nhd1‐Ghd7 regulatory module serves as a molecular integrator that synchronizes photoperiodic and N signaling pathways.

### 2.4 Nhd1 is Required for Ghd7‐Mediated Flowering Inhibition in Long Day

**Figure 3:**
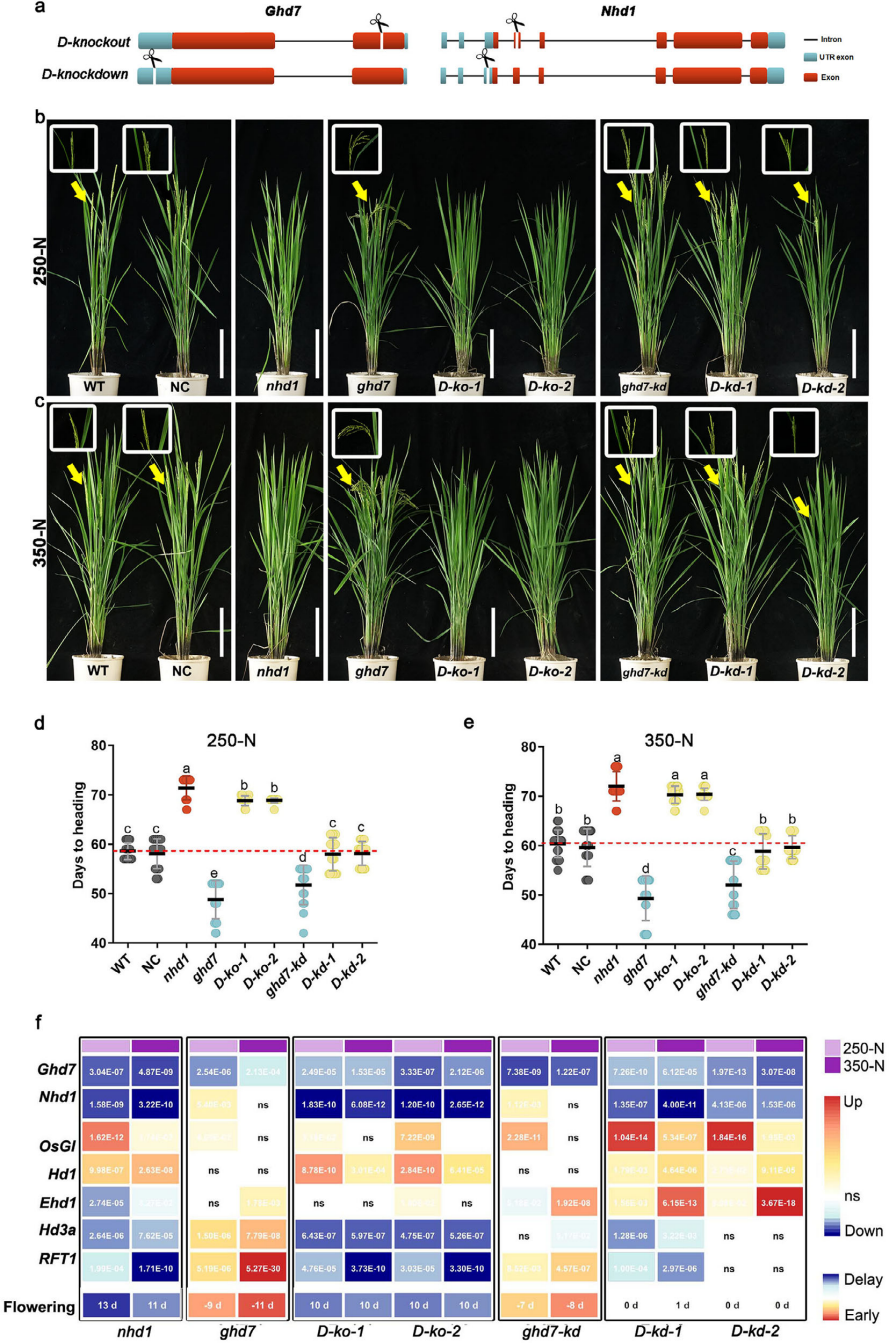
Genetic relationship between Nhd1 and Ghd7 in regulation of flowering time under different photoperiod and N supply conditions. a) Schematic of mutation sites in *Ghd7 and Nhd1* double mutants (indicated by scissors). detail information sees in Figure S3 (Supporting Information). Phenotypes and days to heading of WT, single and double mutants of *ghd7* and *nhd1* supplied with b,d) 250 kg N ha^−1^ and c,e) 350 kg N ha^−1^ in the field under natural long day (NLD) condition (Nanjing, Jiangsu). NC, transgenic negative control; *D‐ko*, double knockout mutant of *nhd1* and *ghd7*; *D‐kd*, double knockdown mutant of *nhd1* and *ghd7*. Scale bar, 20 cm. The arrows and the amplified frames highlight representative spikes. Values are means±SD (*n*≥10). One‐way ANOVA was used for the statistical analysis (*p* ≤ 0.05). f) The differential expression of key flowering genes and the corresponding changes in flowering time in single or double mutants of *Ghd7* and *Nhd1* relative to that in WT under 250  or 350 kg N ha^−1^ N conditions. The fully expanded youngest leaf was collected in the morning for genes expression analysis. The relative value of gene expressions was calculated by [value in mutant/value in WT − 1] × 100%. The *p*‐values (significance of gene expression changes) and the differences of flowering time (in days) between mutants and WT were shown in the boxes. One‐way ANOVA was used for the statistical analysis (*p* ≤ 0.05).

To elucidate the genetic relationship between *Nhd1* and *Ghd7*, we generated two distinct types of *ghd7/nhd1* double mutants (**Figures** **3**a and S3a, Supporting Information). The first type is double knockout of *Nhd1 and Ghd7* (*D‐ko*), containing exonic mutations, which caused frameshifts and complete loss of their functions. The second type is double knockdown of *Nhd1* and *Ghd7* (*D‐kd*), featuring 5′UTR mutations, which reduced transcript levels without altering their protein functions. A *ghd7‐kd* single mutant with a 5′UTR mutation served as a control (Figure S3a, Supporting Information).

Field experiments under natural long‐day (NLD) and short‐day (NSD) conditions with varying N treatments revealed that *nhd1* mutants showed delayed flowering time across all conditions (Figure 3b–e; Figure S4a,b, Supporting Information), whereas *ghd7* mutants showed earlier flowering time only under NLD (Figure 3b–e). Notably, *D‐ko* exhibited flowering delays similar to *nhd1* single mutants under NLD (Figure 3b–e), demonstrating *Nhd1*'s epistasis over *Ghd7* in flowering regulation in LD. This further supports Nhd1's dominant role over Ghd7 in regulating flowering through transcriptional control. In addition, knockdown of *Ghd7* (*ghd7‐kd* mutant) resulted in a moderate promotion of flowering time (Figure 3d,e). However, when both *Nhd1* and *Ghd7* were knocked down, flowering time returned to WT levels (Figure 3d,e; Figure S4a,b, Supporting Information). RNA‐seq analysis and qPCR validation of flowering‐related genes revealed that both *nhd1* and *D‐ko* showed similar significant alteration in floral genes expressions compared to WT, while *ghd7, ghd7‐kd and D‐kd* exhibited only minor changes (Figure 3f; Figure S5a, Supporting Information). This further supports Nhd1's dominant role over Ghd7 in regulating flowering through transcriptional control.

### 2.5 N Supply Dictates the Effects of Nhd1 Promotion and Ghd7 Inhibition on Flowering Time

**Figure 4:**
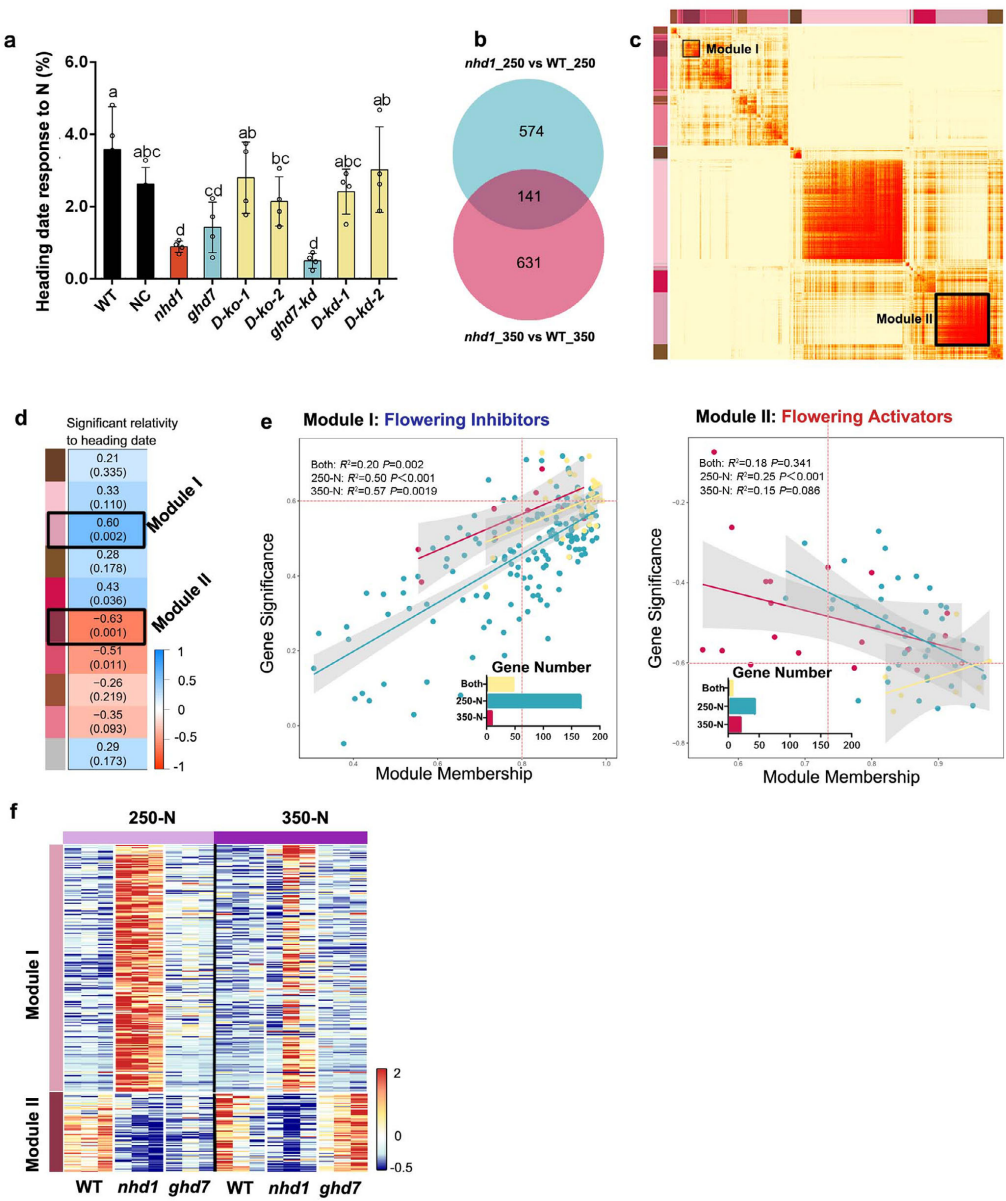
Transcriptional profiling reveals that both Nhd1 activation and Ghd7 suppression to flowering are dependent on N supplies. a) Heading date (flowering time) response to N (%) of WT and mutants, which was calculated as the percentage value of flowering time delayed by increased N supply: [(flowering time at 350 kg N ha^−1^− flowering time at 250 kg N ha^−1^) / flowering time at 250 kg N ha^−1^] × 100%. b) The number of the different expression genes (DEG) between *nhd1* mutant and WT at 250  and 350 kg N ha^−1^. c) The heatmap plot of the topological overlap‐based dissimilarity with hierarchical clustering dendrograms for *nhd1* (see the WGCNA described in materials and methods). The transformation from yellow to red color presents low to high topological overlap. d) The correlation of the eigengene of each module shown in (c) with heading date, with red colors for negative correlations (earlier heading) and blue colors for positive correlations (later heading). Texts within the heatmap indicate the correlation coefficients and the corresponding *p*‐values (in parentheses). e) Scatterplots of genes in module I‐II significance for heading date versus module membership in the most significant modules across N levels. Genes significant represents the correlation between a gene and heading date. Module membership indicates the correlation between an individual gene and the corresponding module eigengene in (d). f) The Transcription profiling of genes in module I and II in WT, *nhd1* and *ghd7* under 250 and 350 kg N ha^−1^ conditions. One‐way ANOVA was used for the statistical analysis in (a,b) (*p* ≤ 0.05).

To assess flowering sensitivity to N, we calculated flowering time ratios between 350 N and 250 N conditions in NLD (Figure 3d,e). WT plants showed a flowering time delayed by 4% under high N (**Figure** **4**a), while both *nhd1* and *ghd7* single mutants exhibited reduced sensitivity by 0.8–1.2% (Figure 4a). Notably, the flowering time of *nhd1* mutants is 13 d later than that of WT at 250‐N and 11 d later at 350‐N (Figure 3f; Figure S5b,c, Supporting Information). These delayed flowering phenotypes correlated with more significant up‐ and down‐regulation of *OsGI* (flowering inhibitor) and *Hd3a* (flowering promoter) in *nhd1* mutant at 250 N than 350 N (Figure 3f). Conversely, the flowering time of *ghd7* mutants is 9 days earlier than that of WT at 250 N and 11 d earlier at 350‐N (Figure 3f; Figure S5b,c, Supporting Information), coinciding with stronger upregulation of *Ehd1*, *Hd3a* and *RFT1* at 350 N than that at 250 N (Figure 3f). These results suggest that the function of Nhd1 and Ghd7 in flowering regulation is distinct in different N conditions.

To elucidate the global regulatory patterns of Nhd1‐ and Ghd7‐mediated flowering time control under different N conditions, we analyzed differentially expressed genes (DEGs) from *nhd1* and *ghd7* mutants (Figure 4b and Figure S5d, Supporting Information). Weighted gene correlation network analysis (WGCNA) of *nhd1*‐associated DEGs identified over 10 distinct co‐expression modules (separated color band on left of Figure 4c). Among these, Module I and Module II exhibited significant correlations with flowering time (heading date) (Figure 4d). Genes in Module I showed positive correlations between genes expression and heading date, suggesting these genes’ role in flowering inhibition, while genes in Module II displayed negative relationships between genes expression and heading date, implicating their roles in flowering activation (Figure 4e). Notably, knockout of *Nhd1* preferentially upregulated Module I (inhibitors) and downregulated Module II (activators) genes at 250‐N, but not at 350 N (Figure 4f). This result aligns with the observed stronger flowering delay in *nhd1* mutants at 250  versus 350 kg N ha^−1^ (Figure 4a), implying the stronger function of Nhd1 in regulating flowering related genes in relatively low N condition.

Similarly, WGCNA of *ghd7*‐associated DEGs identified 9 co‐expression modules (Figure S5e, Supporting Information), of which only Module III showed a significant correlation with flowering time (Figure S5f, Supporting Information). Genes in this module exhibited negative expression‐heading date relationships, suggesting their potential roles in flowering activation (Figure S5g, Supporting Information). However, in the early‐flowering *ghd7* mutant, nearly all Module III genes were downregulated at 250 kg N ha^−1^ and remained unchanged at 350 kg N ha^−1^ (Figure S5h, Supporting Information), implying they are not direct downstream genes in Ghd7‐regulated flowering pathway. These findings further support the conclusion that Ghd7 controls flowering primarily through the Nhd1‐dependent pathway.

### 2.6 Nhd1 and Ghd7 Orchestrate the U‐Shaped N Response of Flowering Time Mainly via Modulating Expression of *Hd3a*

**Figure 5:**
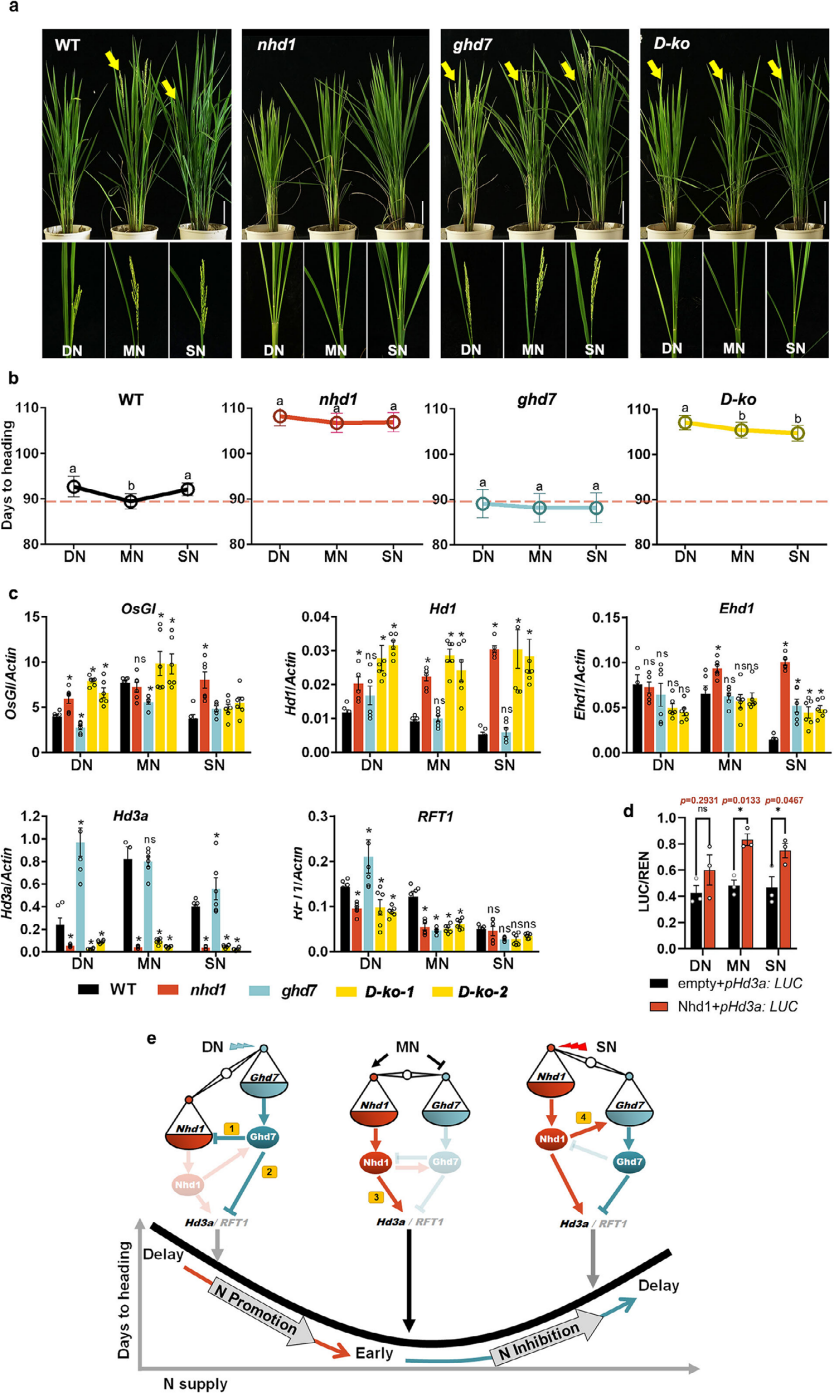
Nhd1, Ghd7 and Hd3a are required for the U‐shape response of flowering time to the supply of N at broad range. a) Flowering phenotypes of WT, *nhd1*, *ghd7* and *D‐ko* mutants growing in paddy field under different N supply levels. Deficient N (DN), moderate N (MN) and superior N (SN) treatments represent supply of 75, 150, and 250 kg N ha^−1^, respectively. White bar indicates 10 cm. The arrows and the amplified frames indicate spikes. b) Days to heading of the plants under different N conditions presented in (a). Values are means±SD (*n* ≥ 30). One‐way ANOVA was used for the statistical analysis (*p* ≤ 0.05). c) Relative expression of *OsGI*, *Hd1*, *Ehd1*, *Hd3a* and *RFT1* under different N conditions in the plants presented in (a). The fully expanded youngest leaf was collected in the morning. Values are means±SE (*n* ≥ 3). d) Transactivation of *Nhd1* on the *Hd3a* promoter in rice protoplast at different N conditions. Student's *t* text is used for the statistical analysis in (c,d) (*p* ≤ 0.05). e) The integrative model in which Nhd1, Ghd7 and florigens (Hd3a and RFT1) coordinate flowering time responses to N availabilities through a “seesaw effect.” Under DN condition, transcripts of *Ghd7* are induced, delaying flowering by directly repressing *Nhd1* expression (pathway 1) and indirectly repressing florigens expression (pathway 2;^[^ ^12^ ^]^); Under MN condition, Ghd7‐mediated repression of *Nhd1* is impaired, allowing Nhd1 to induce florigens expression and promote flowering (pathway 3;^[^ ^9^ ^]^); Under SN condition, the upregulated Nhd1 directly activates *Ghd7* expression (pathway 4), which suppresses florigens genes expression, resulting in delayed flowering time. This dynamic regulatory network produces a characteristic U‐shaped flowering pattern (“delay‐early‐delay”) across varying N availability. The genetic regulation of these pathway is presented in Figure S6b (Supporting Information).

To systematically investigate how Nhd1 and Ghd7 coordinate N‐dependent flowering time regulation, we analyzed their roles across a gradient of N availability in both paddy fields and hydroponic systems. Expression of three key N‐responsive genes^[^ ^37^, ^38^, ^39^ ^]^ was analyzed to characterize the difference among three N conditions in the paddy field (Figure S6c, Supporting Information). Plant phenotypes showed that WT exhibited the characteristic U‐shaped flowering response to N availability in both growth systems, showing delayed flowering under both deficient‐N (DN) and superior‐N (SN) conditions (Figure 5a,b; Figure S6a, Supporting Information). Whereas this U‐shape response was eliminated in both *nhd1* and *ghd7* mutant. Specifically, inactivation of *Nhd1* delayed flowering time across all N regimes, with a more delay days observed in MN conditions (Figure 5a,b, Supporting Information). In contrast, inactivation of Ghd7 promoted flowering time only under DN and SN conditions, while the *ghd7* mutant exhibits flowering time comparable to WT under MN condition (**Figure** **5**a,b).

Interestingly, although the expressions of multiple flowering‐related genes were affected by N supplies or loss‐of‐function of *Nhd1* and *Ghd7*, only the expression of *Hd3a* was consistent with the observed changes in flowering time both in different N conditions and mutants (Figure 5b,c; Figures S6a,d,e and S7, Supporting Information). *Hd3a* is the direct target of Nhd1.^[^ ^9^ ^]^ Transactivation assay further confirmed that activation of *Hd3a* transcription by Nhd1 is stronger in MN condition (Figure 5d). These evidences corroborate our previous finding that Hd3a serves as the terminal flowering regulator activated by Nhd1 in response to N.^[^ ^9^ ^]^ In conclusion, Nhd1 and Ghd7 form an antagonistic feedback regulatory module that monitor transcription of flowering genes, particularly *Hd3a*, in different N conditions, to orchestrate U‐shaped flowering response to N availability (Figure 5e).

### 2.7 Selection of Functional *Nhd1* and *Ghd7* Alleles Fine‐Tunes Flowering Time to Adapt to N Availability in Weak/None Alleles of *Ghd7*

**Figure 6:**
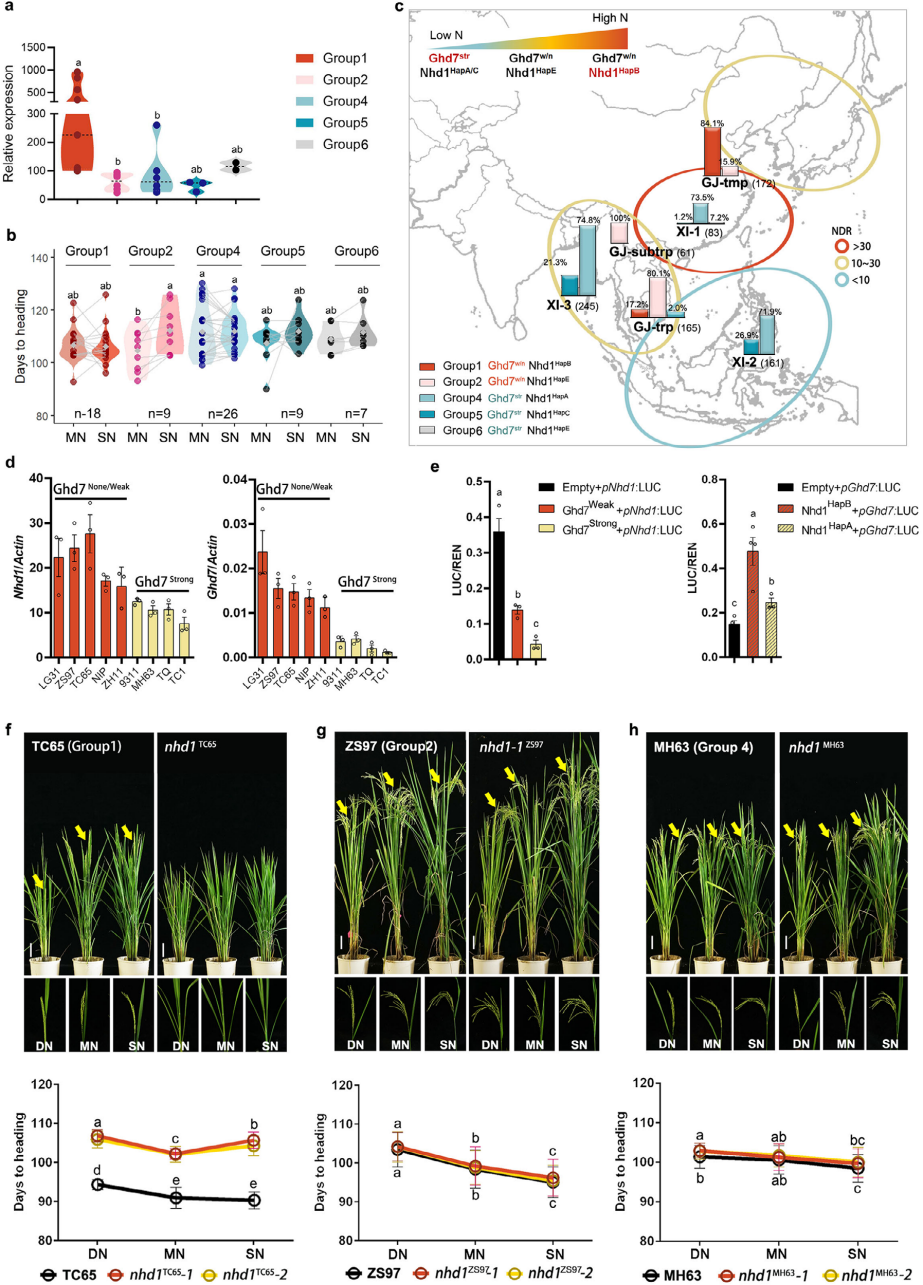
Fine‐tuning N sensitivity of flowering time by selecting different functional Nhd1 and Ghd7 in natural rice cultivars. a) Relative expression of *Nhd1* in rice varieties from different *Ghd7*‐*Nhd1* combination groups. b) Days to heading of rice varieties in different *Ghd7*‐*Nhd1* combination groups under MN (150 kg N ha^−1^) and SN (250 kg N ha^−1^) conditions. Complete flowering time data for all varieties are provided in Table S2 (Supporting Information). (c) Geographical distribution of the *Ghd7*‐*Nhd1* combination groups frequency among different rice subspecies in different soil N deposition rate (NDR; kg ha^−1^ per year) areas in Asia. Regions with different NDR as indicted are roughly divided based on previous studies.^[^ ^31^, ^42^ ^]^ One‐way ANOVA was used for the statistical analysis (*p* ≤ 0.05). d) Expression of *Nhd1* and *Ghd7* in different rice cultivars carrying weak/none or strong functional *Ghd7* alleles. Values are means±SE (*n* ≥ 3). e) Transactivation of different functional Ghd7 and Nhd1 on *Nhd1* and *Ghd7* promoters, respectively, in rice protoplasts. Values are means±SE (*n* ≥ 3). Flowering phenotypes and days to heading of *nhd1* mutants generated in the f) TC65, g) ZS97, and h) MH63 cultivars backgrounds under different N supplies. Scale bar, 10 cm. The arrows and the amplified frame indicate spikes. Values are means±SD (*n* ≥ 30). One‐way ANOVA was used for the statistical analysis in (d–h) (*p* ≤ 0.05).

To investigate the evolutionary conservation of the Nhd1‐Ghd7 regulatory module, we analyzed allelic variation patterns across rice genotypes using 3K genome data.^[^ ^40^ ^]^ *Ghd7* exists as strong, weak and nonfunctional alleles, showing distinct subspecies distributions between *indica* (mainly *Ghd7* ^str^) and *japonica* (mainly *Ghd7* ^w/n^).^[^ ^29^, ^41^ ^]^ Seven main groups are identified by combining different functional Ghd7 alleles and *Nhd1* promoter haplotypes (Figure S8a—c, Supporting Information;^[^ ^9^ ^]^). Among them, the *Japonica* groups (group 1: Ghd7^w/n^ with Nhd1^HapB^) showed much higher *Nhd1* expression than *indica* groups (group 4‐6: Ghd7^str^ with *Nhd1* ^HapA/C/E^) (**Figure** **6**a; Figure S8a—c, Supporting Information), while group 2 suppressed *Nhd1* expression compared to group 1, although they share the same *Ghd7* allele (*Ghd7* ^w/n^) (Figure 6a; Figure S8a—c, Supporting Information). Interestingly, group 1 showed insensitivity of flowering time to N supplies, while group 2 returned this sensitivity (Figure 6b). The results suggest that the natural variation of *Nhd1* in its promoter SNPs outside the Ghd7‐binding EEL element (Figure 1i) can modulate flowering time regulation independently of Ghd7 functionality.

Both *japonica* and *indica* subspecies are widely cultivated across Asia under varying N conditions.^[^ ^31^, ^42^ ^]^ While *japonica* typically carries *Ghd7* ^w/n^ alleles and *indica* harbors *Ghd7* ^str^ alleles, their combinations with different *Nhd1* haplotypes show remarkable variability in flowering time and its response to N (Figure 6b). To examine potential adaptation links, we analyzed the combinatorial allele distributions relative to regional N deposition rates (NDR).^[^ ^42^ ^]^ *Indica* rice with different genotypes (mainly groups 4‐6) showed balanced distribution across NDR gradients (Figure 6c), while *japonica* rice (mainly group 1–2) was isolated by genotypes in different NDR. 84.1% of group 1 (temperate *japonica*) predominated in high‐NDR (>30 kg ha^−1^) regions, while group 2 (subtropical/tropical *japonica*) dominated in lower‐NDR (10–30 kg ha^−1^) zones (Figure 6c). This distribution pattern supports our finding that *Nhd1* expression primarily regulates N‐responsive flowering in *Ghd7* ^w/n^ backgrounds, suggesting adaptive selection of *Nhd1* haplotypes based on local N availability in weak/none alleles of *Ghd7*.

Our previous work has identified four SNPs in coding sequence of different *Nhd1* haplotypes.^[^ ^9^ ^]^ One of these SNPs at position 1457 bp (C→T) results in a amino acid substitution, distinguishing Ser^486^ in *Nhd1* ^HapA/C/D/E^ from Phe^486^ in *Nhd1* ^HapB^ (Figure S8d, Supporting Information). This residue change may introduce a potential phosphorylation site for CK2 (Casein kinase II)^[^ ^43^ ^]^ and confer unique biochemical and functional properties of Nhd1^HapB^ protein from other haplotypes (Figure S8e, Supporting Information). Expression of both *Nhd1* and *Ghd7* were both more amplified in *Ghd7* ^w/n^ alleles (Figure 6d). Transcriptional assays further revealed that the Ghd7^weak^ protein weakly suppresses *Nhd1* expression, while the Nhd1^HapB^ protein in *Ghd7* ^w/n^ backgrounds significantly activates *Ghd7* transcription (Figure 6e). To characterize the function of different Nhd1 haplotypes in flowering regulation, we generated *Nhd1* mutants in three representative cultivars: TC65 (*Ghd7* ^weak^ *Nhd1* ^HapB^), ZS97 (*Ghd7* ^none^ *Nhd1* ^HapE^), and MH63 (*Ghd7* ^str^ *Nhd1* ^HapA^). *K*nockout of *Nhd1* in TC65, with decreased expression of florigens (Figure S9a, Supporting Information), significantly delayed flowering across three N regimes (Figure 6f), but showed minimal effect in ZS97 and MH63 (Figure 6g,h; Figure S9b,c, Supporting Information), demonstrating the predominant role of *Nhd1* ^HapB^ in flowering regulation in *Ghd7* ^w/n^ backgrounds, rather than in *Ghd7* ^str^ backgrounds. These findings reveal the haplotype‐specific regulatory hierarchy between Nhd1 and Ghd7.

## 3 Discussion

### 3.1 Antagonistic Module of Nhd1‐Ghd7 Regulates Flowering Time Adapt to N Availability

Nhd1 and Ghd7 have been characterized as critical regulators in photoperiod pathway.^[^ ^34^, ^41^ ^]^ The transcriptional interplay between these two factors fine‐tunes flowering time in responding to different N conditions. Nhd1 (OsCCA1/OsLHY) has been characterized as a flowering promoter.^[^ ^9^, ^34^, ^36^ ^]^ *Nhd1* expression is impaired in deficient‐N condition, which turns to suppressed expression of *Hd3a* and results in delay of flowering time.^[^ ^9^ ^]^ In this study, we provide several lines of evidence the suppression of *Nhd1* under DN conditions is primarily caused by the high accumulation of Ghd7. First, Ghd7 can directly bind to the promoter of *Nhd1* to suppress its expression (Figure 1). The second, the inhibitory effect of Ghd7 on *Nhd1* expression is much stronger at DN condition (Figure 2e,f). The third, the expression of *Ghd7* is activated by DN (Figures 1f and 2d), and inactivation of *Ghd7* alleviated the DN induced suppression of *Nhd1*, leading to comparable abundance of *Nhd1* transcripts across different N conditions (Figure 2f). The fourth, additional knockdown of *Nhd1* in *ghd7* knockdown mutant (*D‐kd*) completely restored flowering time to WT levels (Figure 3b–e), demonstrating that the suppression of *Nhd1* transcription by activated Ghd7 results in the DN induced delay of flowering.

Moreover, *Nhd1* is up‐regulated by increasing N supply (Figure 1e ^[^ ^9^ ^]^), while the flowering time is postponed at superior N condition (350 kg N ha^−1^ in Figure 1a–d). This contradict functions of Nhd1 in regulating flowering time can be explained by the enhanced expression of *Ghd7* by Nhd1 at SN. Under SN condition, Ghd7 loss its repression on *Nhd1* expression (Figure 2k,l), while the upregulated‐Nhd1 significantly induced transcription of *Ghd7* (Figure 2h–i). Knockout of *Nhd1* dramatically decreased the expression of *Ghd7* at SN (Figure 2c,d and Figure S3b, Supporting Information), suggesting Nhd1 is required for maintaining *Ghd7* transcription at SN. In fact, the abundance of *Ghd7* transcripts of rice grown in paddy field was indeed more abundant at higher N regime (Figure S3b, Supporting Information). Interestingly, Gln significantly induced expression of *Ghd7*, which required Nhd1 (Figure 2c,h), revealing that SN with high level of Gln upregulates the expression of *Ghd7* in a Gln‐Nhd1 mediated manner. However, *Ghd7* expression was also upregulated when N supply is deficient (Figures 1f and 2d), where function of Nhd1 is weaken, indicating other regulators involving in mediating *Ghd7* expression at DN. Nevertheless, in view of the earlier flowering phenotype of *ghd7* mutants at both DN and SN (Figure 5a,b), it can be concluded that Ghd7 contributes to not only the low‐ but also the high‐ N induced inhibition of flowering time.

### 3.2 Hd3a Acts as a Central Integrator of N‐Regulated Flowering Time Control in Rice

Hd3a and RFT1 are both florigens, serving as a hub in flowering regulation.^[^ ^41^ ^]^ Whereas Hd3a, rather than RFT1 dominates N‐regulated flowering time. Expression of *Hd3a*, not *RFT1*, is consist to flowering time in WT and mutants grown in different N conditions (Figure 5 and Figure S9, Supporting Information). Our previous work has revealed that Nhd1 directly activates *Hd3a* to promote flowering.^[^ ^9^ ^]^ In this study, we further demonstrated that Nhd1 regulation of *Hd3a* transcription is N‐dependent. The direct activation of *Hd3a* by Nhd1 is much stronger at MN than that at DN and SN (Figure 5d), resulting in the earlier flowering time at MN (^[^ ^41^ ^]^; Figure 5e). However, knockout of *Nhd1* also suppressed *Hd3a* at DN and SN (Figure 5c), indicating that the other factors are involved in the Nhd1 regulation of *Hd3a* transcription. It is known that Hd1, a direct suppressor of *Hd3a* transcription, is required for the regulation of Nhd1 in flowering.^[^ ^12^, ^36^ ^]^ Interestingly, we found that, *Hd1* expression was increased in *nhd1* mutant irrespective of N levels (Figure 5c). The flowering time of *nhd1*/*hd1* double mutant is similar to that of the *hd1* single mutant (Figure S6d, Supporting Information), indicating that Hd1 is epistasis to Nhd1 in the promotion of flowering. Although Nhd1 cannot directly regulate *Hd1* transcription,^[^ ^34^ ^]^ these results suggest that Nhd1 can also indirectly regulate *Hd3a* transcription through other regulators, potentially via Hd1 or Ghd7‐Hd1 dependent pathways.

In contrast to Nhd1, the *ghd7* mutant shows upregulated *Hd3a* expression and earlier flowering specifically at DN and SN (but not MN) conditions (Figure 5a–c), suggesting the role of Hd3a in Ghd7‐mediated flowering inhibition under DN and SN regimes. Ghd7 mediates flowering delay through dual mechanisms: suppressing *Nhd1* expression via transcription regulation (Figure 2e,f, k,l), while also inhibiting Hd1 transactivity via protein interaction (^[^ ^12^, ^36^ ^]^; Figure S6b, Supporting Information). These results collectively identify that Hd3a is the central integrator that transmits the N signal perceived by the Nhd1‐Ghd7 module to regulate flowering time under different N availability.

Moreover, although the expression of *Hd3a* remains correlated with flowering time in other rice cultivars, the induction of *Hd3a* is uncoupled from Nhd1 regulation in cultivars carrying weak alleles of *Nhd1* (Figure S9, Supporting Information). These results indicate the existence of other regulators modulating N‐mediated flowering time in the absence of Nhd1 function.

The discovery of this regulatory module not only provides a molecular explanation for field observations of N‐responsive flowering variation, but also reveals how rice maintains developmental stability under optimal N conditions while retaining plasticity to adjust flowering time across varying N environments, representing a sophisticated adaptation mechanism that coordinates reproductive timing with N availability.

### 3.3 Allelic Selection of *Nhd1* and *Ghd7* Underlies the Variation of N‐Adaptive Flowering Time in Rice Domestication

Photoperiod serves as the primary determinant of flowering time in plants, while N acts as a fine‐tuning modulator under constant photoperiodic conditions. As demonstrated in the previous studies, natural allelic variations of *Ghd7* contribute to photoperiod sensitivity performances in rice domestication,^[^ ^41^ ^]^ while the *Ghd7* alleles alone can't fully explain the distribution of the groups in different NDR showing in Figure 6C. This conclusion is based on the following evidences. First, we found two combination groups (1 and 2) which carry the same *Ghd7* ^weak/none^ alleles, but different *Nhd1* alleles (Figure S8c, Supporting Information), displaying the distinct flowering response to N supplies (Figure 6b), suggesting that Ghd7 is not the critical factor that regulates the N sensitivity of flowering time in these groups. Second, the *Ghd7* ^w/n^ *Nhd1* ^HapB^ haplotype (group 1) exhibits high *Nhd1* expression and predominance in optimal‐to‐high NDR areas, while the *Ghd7* ^w/n^ *Nhd1* ^HapE^ haplotype (group 2) exhibits low *Nhd1* expression and distributes in low‐to‐optimal NDR areas (Figure 6a,c). It is clear that the selection of functional *Nhd1* alleles fine‐tunes flowering time to adapt to N availability in weak/none alleles of *Ghd7*. Third, knockout of *Nhd1* ^HapB^ results in delayed flowering time, while knockout of *Nhd1* ^HapE^ does not significantly affect flowering time (Figure 6). These results indicate that the priority of natural variation of *Nhd1* in the regulation of flowering time under varied N supply conditions in the absence of *Ghd7* function.

Meanwhile, the antagonistic Nhd1‐Ghd7 regulatory module underwent differential selection to optimize flowering time across diverse N environments during rice domestication. Nhd1 and Ghd7 exhibit opposing effect on multiple agronomic traits beyond flowering, including drought tolerance, tiller number, yield and N utilization (Figures S1 and S10a, Supporting Information;^[^ ^9^, ^29^, ^44^, ^45^ ^]^). Knockout of *Ghd7* promoted flowering time and decreased yield (Figure S10a, Supporting Information^[^ ^29^ ^]^), while knockout of *Nhd1* delayed flowering time and increased both yield and NUE (Figure S10, Supporting Information^[^ ^9^ ^]^). Intriguingly, we found that knockout of *Nhd1* in the cultivar (ZS97) carrying non‐functional alleles of *Ghd7* did not affect the flowering time (Figure 6g), yield and NUE (Figure S10c, Supporting Information). However, knockout of *Nhd1* in the cultivar (MH63) carrying strong functional alleles of *Ghd7* did not alter flowering time (Figure 6h), but increased both yield and NUE very significantly at low N supply (Figure S10d, Supporting Information). Such improvement of rice yield and NUE might be due to the enhanced N assimilation and translocation caused by inactivation of *Nhd1*.^[^ ^9^, ^46^, ^47^ ^]^ These results demonstrate that the function of Nhd1 in regulating flowering time and NUE relies on the function of *Ghd7*.

Optimizing the growth duration with high yield is main target for rice breeding.^[^ ^44^ ^]^ Our finding suggests that the combination of elite alleles of *Nhd1* and *Ghd7* (like the cultivar MH63) is a potential approach to develop stress‐resilient, high yield and NUE rice varieties without extending the growth period (flowering time).

## 4 Experimental Section

### Plant Materials

The *nhd1* mutants were the same as those used in our previous work.^[^ ^9^ ^]^ The *ghd7‐1* (*ghd7*), *ghd7‐2*, and *ghd7*/*nhd1* double mutants used in this study were generated by CRISPR/Cas9 system with Nipponbare (*O. sativa* L. ssp. *japonica*) as background. The spacers library of whole rice genome (http://crispr.hzau.edu.cn/CRISPR/) was used for choosing mutation sites. Two types of *ghd7* mutants were generated using guiding spacer in different structure of *Ghd7* sequences. Spacer (CCGAGTGCGTGCCAGGGGAT) of the first type mutant of *ghd7* (*ghd7‐1*) is in the second extron of *Ghd7*. The spacer (GCTATAGCTAACTTACTAGC) of the second type mutant of *ghd7* (*ghd7‐2*) is in the 5′ UTR region of *Ghd7*. For loss‐of‐function *ghd7*/*nhd1* double mutant (*g7n1*‐1 and *g7n1‐2*), the spacers in the second extron of *Ghd7* (spacer: TCCACCTATGTCGATCCTAG) and *Nhd1* (spacer: CCTGGCAGCGCATAGAAGGTGAA) were used. For knockdown double mutants (*g7n1‐3* and *g7n1‐4*), spacer in the 5′ UTR region of *Ghd7* (spacer: GCTATAGCTAACTTACTAGC), and *Nhd1* (spacer: GTCAGGTCCAGCGTTTGGT) were used. For *nhd1*/hd1 double mutant (*n1h1*), the spacers in the first extron of *Hd1* (spacer: AACGTGTTCGACCAGGAGGT), and in the second extron of *Nhd1* (the same spacer with *g7n1‐1/2*) were used. Negative control (NC) line had the same transgenic process with mutants but without causing mutation. The detail information of these mutants is shown in Figure S3 Supporting Information.

### Plant Growth Conditions

For hydroponic experiments, plants were grown in a phytotron with a day/night temperatures of 35 °C/24 °C and relative humidity of 65%. The 14 h light/10 h dark cycle was used as the long‐day condition, and the 10 h light/ 14 h dark cycle was set as the short‐day condition. Except N concentration, the IRRI nutrient solution was used as the same as described.^[^ ^48^ ^]^ (NH_4_)_2_SO_4_ was used as N resource for the different N concentration treatments in Figures 1 and 2, and Figure S6 Supporting Information.

For field experiments, plants were grown under long‐day condition in Nanjing, Jiangsu province (119°02′ E‐31°65′ N), with an average of 13.5 h of day time and 10.5 h of night time. Plants were grown under short‐day condition in Ledong, Hainan province (18°45′ N–109°10′ E), with an average of 13.5 h of day time and 10.5 h of night time. Based on local rational N fertilization,^[^ ^49^, ^50^, ^51^, ^52^ ^]^ the total amount of applied N was 250  and 350 kg N ha^−1^ in Figures 1, 3, and 4; Figures S1, S3–S5, and S8 (Supporting Information). Total amount of applied N was 75, 150, and 250 kg N ha^−1^ in Figures 5 and 6 and Figure S6 (Supporting Information). Each line was planted in a plot with an area of 1.68 m^2^ and an arrangement of 7 × 8 plants.

### Flowering Time Recording

In the field experiment, the day to appearing of the first flowering in main panicle of each plant is defined to the heading date (flowering time). The flowering frequency was calculated by dividing the number of flowering plants by the total number of plants each day in a field plot. In the hydroponic experiment, the day to heading of each plant with in the plot was recorded as flowering time.

### The Nitrogen Treatments for Recording the U‐Shape Response of Flowering Time

We provided a range of N treatments in both hydroponic system and paddy field experiments to record flowering time of rice. Moderate‐N (MN) refers to the N condition at the base of the U‐shape curve where WT plant shows the earliest flowering time. Deficient‐N (DN) and superior‐N (SN) correspond to the N conditions on the left and right sides of the U‐shape curve, respectively.

In the hydroponic system, plants were first grown in basal nutrient solution containing 2.5 mm NH_4_^+^) for 30 d, then transferred to four different N treatments, including 0, 0.25, 2.5, and 10 mm until flowering. As the definition above, the 0.25 mm N concentration at which flowering was the earliest, is referred as the moderate‐N condition, meanwhile 0 mm N was defined as deficient‐N; and both 2.5‐ and 10 mm N were defined as superior‐N. Since we supplied the sufficient N (2.5 mm) in the seedling stages until start of the treatments with different N concentrations, therefore, the 0 mm N treatment is recognized as the condition of relative low N rather than limited N.

In the field experiments, four levels of N fertilizers were supplied at the soil: 75, 150, 250 and 350 kg ha^−1^. Since the flowering time at 150 kg ha^−1^ supply was the earliest for the cultivar of Nipponbare, thus, it is referred as moderate‐N level. Meanwhile, 75 kg ha^−1^ and 250–350 kg ha^−1^ were at the left and right side of the U‐shape curve for the flowering time responses to N, they are referred as deficient‐N and superior‐N, respectively.

### Determination Genes Expression in Different Circadian Period and N Conditions

All the plants were cultured with sufficient nutrient solution in a phytotron until five leave stage and transferred to different treatments for analysis.

For diurnal gene expression under different N conditions shown in Figure 1, plants were transferred to N‐free solution for three days before being supplied with 0.25 mm, 2.5 mm or 10 mm N concentration solution for further 3 d. Samples were collected every 4 h at 8:00 am (ZT0) on the fourth day. The fully expanded youngest leaf was collected for the gene analysis.

For diurnal gene expression under different photoperiods shown in Figure 2, plants were transferred to different photoperiod conditions, long‐day (light 14 h/ dark 10 h), short day (light 14 h/ dark 10 h), constant dark and constant light, respectively, for a week before sampling. The fully expanded youngest leaf was collected every 4 h from ZT0.

For genes expression under different N forms and concentrations shown in Figure 2, plants were transferred to N‐free solution for three days and supplied with 0, 0.125, 1.25, or 5 mm (NH_4_)_2_SO_4_ or Gln for 3 d. The fully expanded youngest leaf was collected at ZT4 for analysis.

For diurnal flowering gene expression under different N conditions shown in Figure S7 (Supporting Information), plants were transferred to solution supplying with 0 , 0.125, and 1.25 mm for 15 d. Samples were collected every 4 h at 8:00 am (ZT0). The fully expanded youngest leaf was collected for the gene analysis.

For genes expression under different N forms. Plants were transferred to N‐free solution for 3 d and supplied with different N form solutions (other nutrients are same as basis) for 3 h before sampling. Total N concentration in each treatment was 2.5 mm. 0.1 mm MSX was added to prevent Gln synthesis and additional Gln was used to mimic the effect of MSX. The fully expanded youngest leaf was collected at ZT0 and ZT8 pm since it was the peak‐expression‐time of *Nhd1* and *Ghd7*, respectively.

For flowering genes expression in WT and mutant plants growing in the field. Three individual plants within each plot (or treatment) free from the edges were randomly selected for flowering‐related genes analysis at the 15 d before the WT starting to flower.^[^ ^29^, ^41^, ^53^ ^]^ The fully expanded youngest leaf was collected in the morning.

### Yeast One Hybrid Assay

The yeast‐one‐hybrid assay was performed using Matchmaker Gold yeast One‐Hybrid Library screening System (Clontech, Takara). Full length CDS of *Ghd7* was cloned into *p*GADT7 vector to generate *p*AD‐Ghd7. The *p*AD‐Nhd1 vector and the NBS (Nhd1‐binding‐site) were verified in previous study.^[^ ^9^ ^]^ To construct *Nhd1*/*Ghd7* promoter‐based reporter vectors, the fragments containing EE‐like/NBS in *Nhd1*/*Ghd7* promoter were amplified. Then *p*GADT7 carried with Nhd1 or Ghd7 and *p*AbAi carried with different sequences were co‐transformed into Y1H Gold yeast strain.

### ChIP‐qPCR Assay

The chromatin immunoprecipitation assay was performed using *EpiQuik* Plant ChIP Kit (EpigenTek). Approximately 1 g of the flag blades of transgenic *p*35S:Flag‐Nhd1 and *p*Ubi:Flag‐Ghd7 plants before the flowering stage was harvested into a 50 mL Falcon tube and add 20 mL 1% formaldehyde to cross‐linking for 10 min. After cross‐linking, 1.25 mL of 2 m glycine solution was added and continued vacuum infiltration for an additional 5 min. The sample was washed three times and then removed into a fine powder with liquid nitrogen to be grinded. The DNA of the tissues was sheared by sonication. Anti‐Flag was performed for immunoprecipitation. The DNA was purified and served as template for real time PCR.

### EMSA Assay

The pMBP‐Ghd7 and pHis‐Nhd1 expression vector were transformed into Rosetta 2 strain of *Escherichia coli*. The expression and purification of MBP‐Ghd7 and His‐Nhd1 fusion protein were performed using amylose resin beads (SinoMol) and Ni‐NTA agarose (SinoMo). The complementary oligonucleotides labeled with biotin at the 5′ end was synthesized (Genscript) and annealed. LightShift Chemiluminescent EMSA Kit (Thermo Fisher Scientific) was used to perform following assay.

### Dual‐Luciferase Report Assay

In Figures 1 and 2, the *Ghd7* and *Nhd1* coding sequence were cloned into *p*Green‐SK vector as the effector. The 2992 kb promoter fragments of *Nhd1* and 2926 kb promoter fragments of *Ghd7* were respectively amplified from Nipponbare, and inserted into pGreen‐ll 0800 vector as reporters. The sequences with random mutations in NBS (AAAAgggg, p*Ghd7*m) and EEL (GGGGATTTA, p*Nhd1*m) were used as negative control. The seedings were germinated on 1/2 MS (Murashige and Skoog) medium and grown under 14 h light/10 h dark or constant dark conditions. Rice protoplasts were extracted as described in a previous study^[^ ^54^ ^]^ and infiltrated with combinational vectors for 16 h. For different N treatment, the rice protoplast was treated with 0.25 mm N and 2.5 mm N for 16 h after infiltrating with combinational vectors.

In Figure 6, the effectors generated from Nipponbare and the reporters were used as the same as which in Figures 1 and 2. The *Ghd7^MH63^* and *Nhd1^MH63^* coding sequence from MH63 cultivar were cloned into *p*Green‐SK vector as the other effectors. The seedings from Nipponbare were germinated on 1/2 MS medium for rice protoplasts extraction and infiltration.

### Real‐Time PCR

Total RNAs were extracted from different tissues using Trizol regent (Thermo Fisher Scientific). Full‐length cDNAs were reverse transcribed using HiScript II Q RT Supermix (Vazyme). Real‐time PCR was performed by AceQ qPCR SYBR Green Master Mix (Vazyme). The rice gene *OsActin2* and *OseEF‐1a* were used for internal references.^[^ ^55^, ^56^ ^]^ The primers using for qPCR is listed in Table S1 (Supporting Information).

### RNA‐Seq Analysis

The WT, *nhd1*, *ghd7* and *g7n1* mutants leave were collected on the 15 d before flowering. Total RNA was extracted using trizol (Thermo Fisher Scientific) and *SteadyPure* RNA Extraction Kit (Accurate Biotechnology). RNA‐seq were performed by Wuhan IGENEBOOK Biotechnology (http://www.igenebook.com). DEGs (differentially expressed genes) were identified with edgeR with a filter threshold of FDR<0.05 and |log_2_FoldChange| >1. Feature count (v1.6.0) software was used for transcript abundance estimation and normalization of expression values as FPKM (Fragments per kilobase of transcript per million fragments mapped).^[^ ^57^ ^]^ The Heatmaps is performed by ggplot2 package in R studio software.

### Weighted Gene Correlation Network Analysis

Weighted gene correlation network analysis (WGCNA) was employed to discover highly correlated gene sets, known as modules, based on weighted correlations between gene expressions.^[^ ^58^ ^]^ Before the analysis, gene expression data were normalized using log_2_(FPKM + 1) transformation. The PickSoft threshold function in the WGCNA package was used to determine an appropriate soft thresholding power for constructing a scale‐free network. Subsequently, a topological overlap matrix was calculated and transformed into a dissimilarity measure. Genes were then clustered using average linkage hierarchical clustering based on the dissimilarity. Modules were identified in the resulting dendrogram using the Dynamic Hybrid tree cut method, with a minimum module size of 30. Modules with highly correlated eigengenes were merged at a height of 0.25.^[^ ^58^ ^]^ Eigengenes of modules were calculated to capture the gene expression patterns within each module. These eigengenes were also used to characterize the correlations between modules and flowering time. Genes within the most significant modules were further screened to assess the relationship between gene significance and module membership. These analyses were conducted on nhd1 and ghd7, respectively.

### Calculation of Photoperiod Sensitivity

The general linear models^[^ ^59^ ^]^ were used to remove the effect of N supplies in different places and assess the individual effects of photoperiod on the heading date.

### Protein Structure Prediction and Alignment Analysis

3D protein structure of five Nhd1 haplotypes was predicted using AlphaFold3 (alphafoldserver.com). The PDB format of all the predicted proteins was input to DALI protein structure comparison server (http://ekhidna2.biocenter.helsinki.fi/dali/; ^[^ ^60^ ^]^) for alignment analysis. The Z scores of the alignments were symmetrized by taking the average of the Z‐score matrix with its transpose (see Table S3, Supporting Information). The scores were visualized via correspondence analysis using a multidimensional scaling method.

### Quantification and Statistical Analysis

Data in the field experiments are presented as means ± SD (standard deviation) with the biological replicates of each plant material as indicated in each figure legend. Data for genes expression are presented as means ± SE (standard error of the mean) with three biological replicated and three technical replicates. For details on the statistical methods, see the figure legends.

### Gene Accession Number

Genes in this paper can ben index in the Ensemblplants database (ensembl.gramene.org). The accession number of each gene: *Nhd1* (Os08g0157600), *Ghd7* (Os07g0261200), *OsGI* (Os01g0182600), *Hd1* (Os06g0275000), *Ehd1* (Os10g0463400), *Hd3a* (Os06g0157700), *RFT1* (Os06g0157500).

## Conflict of Interest

The authors declare no conflict of interest.

## Author Contributions

G.X. and S.Z. planed and designed the research. S.Z. and G.S. conducted most of the experiments and data analysis. X.L. conducted the WGCNA analysis. J.G. analyzed the sensitivity of flowering time to N and photoperiod. M.Y. provides the rice accessions for different haplotypes analysis. Y.J., X.S., and W.D. conducted parts of field experiments. S.Z. and G.X. wrote the manuscript. All authors helped the field experiments and contributed to the finalization of the manuscript.

## Supporting information



Supporting Information

Supporting Tables

Supporting Data

Supporting Animation

## Data Availability

The data that support the findings of this study are available in the Supporting Information of this article.
