# Supplementary material for: U‐Shaped Response of Flowering Time to Low and High Nitrogen via a Transcription Feedback Loop in Rice
Source: Adv Sci (Weinh). 2025 Dec 7;13(4):e08498. doi: 10.1002/advs.202508498 (PMC12822397; doi:10.1002/advs.202508498)
Supplement: Supplementary file 1 — Supporting Information [file ADVS-13-e08498-s003.pdf]

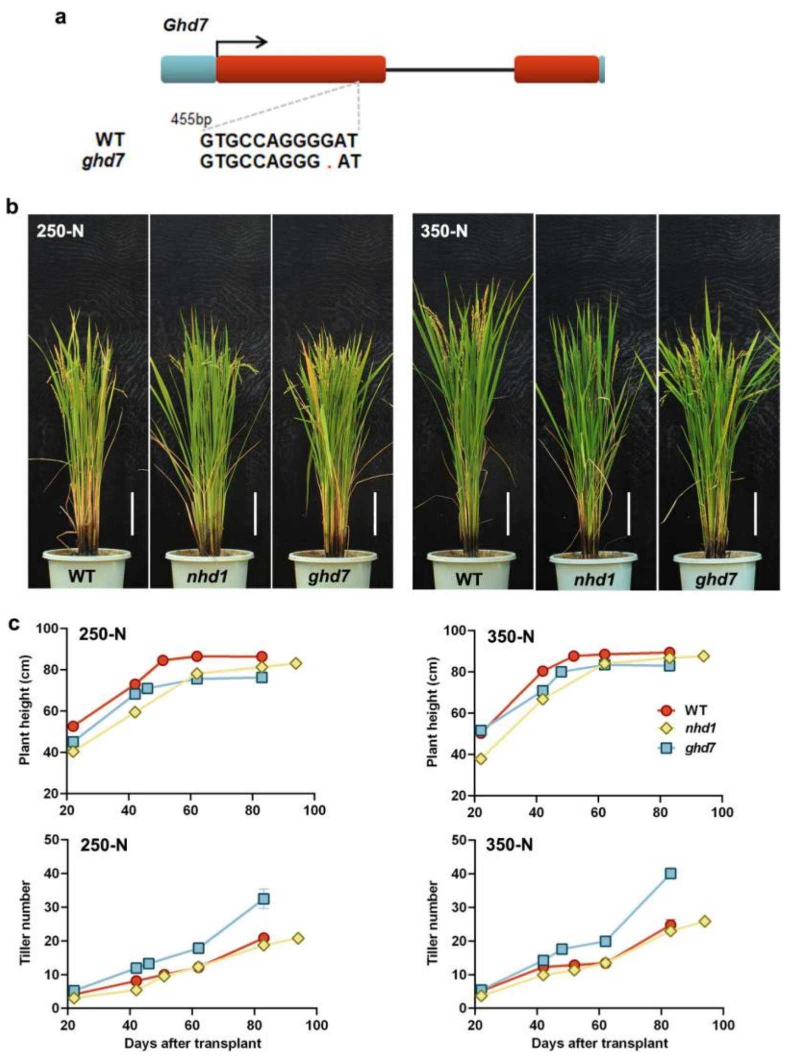

Supplemental figure 1. The dynamic changes of plant height and tiller number of WT, *nhd1* and *ghd7* mutants during entire growth period.

(a) Mutation site of *ghd7* mutant in *Ghd7* gene structure. (b) Photographs of WT, *nhd1* and *ghd7* in mature stage under 250 kg-N/ha and 350 kg-N/ha conditions. Scale bar indicates 20 cm. (c) Dynamic changes in plant height and tiller number of indicating plants from 20 days after transplanting to the mature stage. The *nhd1* mutant has longer growth period than WT and the *ghd7* mutant. Values are means±SD (n⩾10).

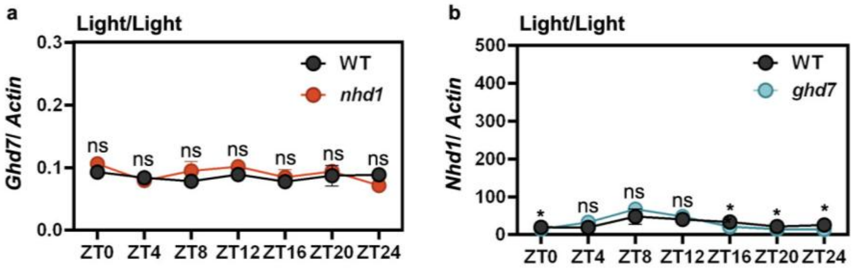

Supplemental figure 2. Relative expression of *Nhd1* and *Ghd7* in WT, *nhd1* and *ghd7* mutants under constant light condition.

(a) Relative expression of *Ghd7* in WT and *nhd1* mutants under constant light condition.

(b) Relative expression of *Nhd1* in WT and *ghd7* mutants under constant light condition.

Values are means±SE (n⩾5). Student's *t* text is used for the statistical analysis ($p \leqslant 0.05$).

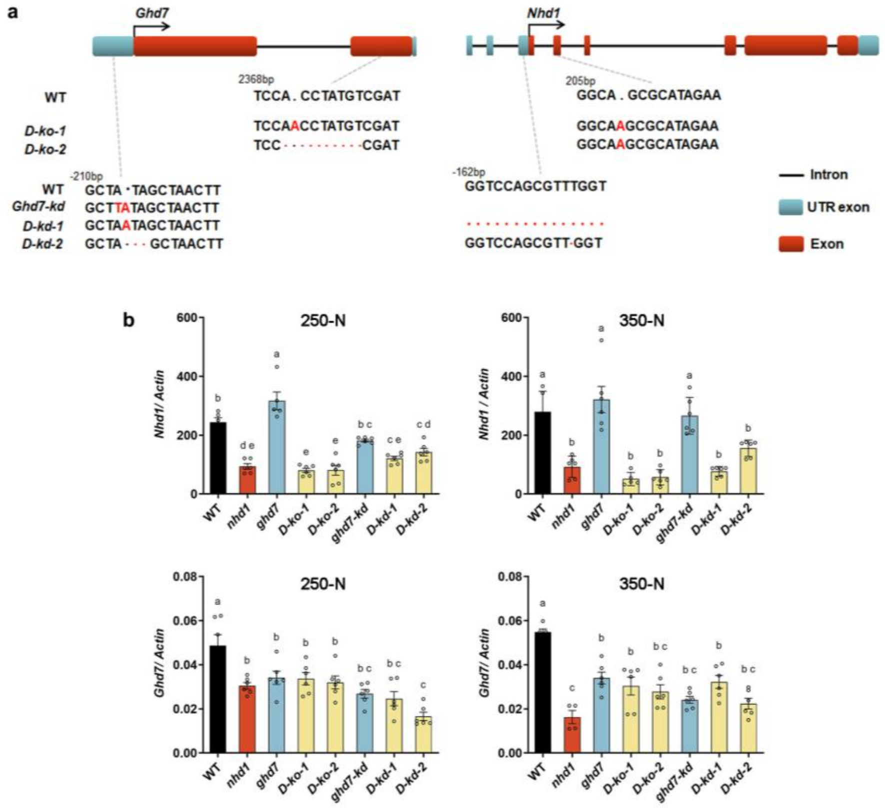

Supplemental figure 3. Schematic diagram of mutation in *ghd7* single mutants and *ghd7/nhd1* double mutants.

(a) Mutation sites of knockdown of *Ghd7* mutant (*ghd7-kd*), double knockout (*D-kd*) and knockdown (*D-ko*) of *ghd7* and *nhd1* mutants. The red letters and dots represent the insert or deletion in the reference sequences of *Ghd7* or *Nhd1* genes. (b) Relative expression of *Nhd1* and *Ghd7* in WT, single and double mutants of *nhd1* and *ghd7* under 250 kg-N/ha and 350 kg-N/ha conditions. Values are means±SE (n⩾5). One-way ANOVA was used for the statistical analysis ($p \leqslant 0.05$).

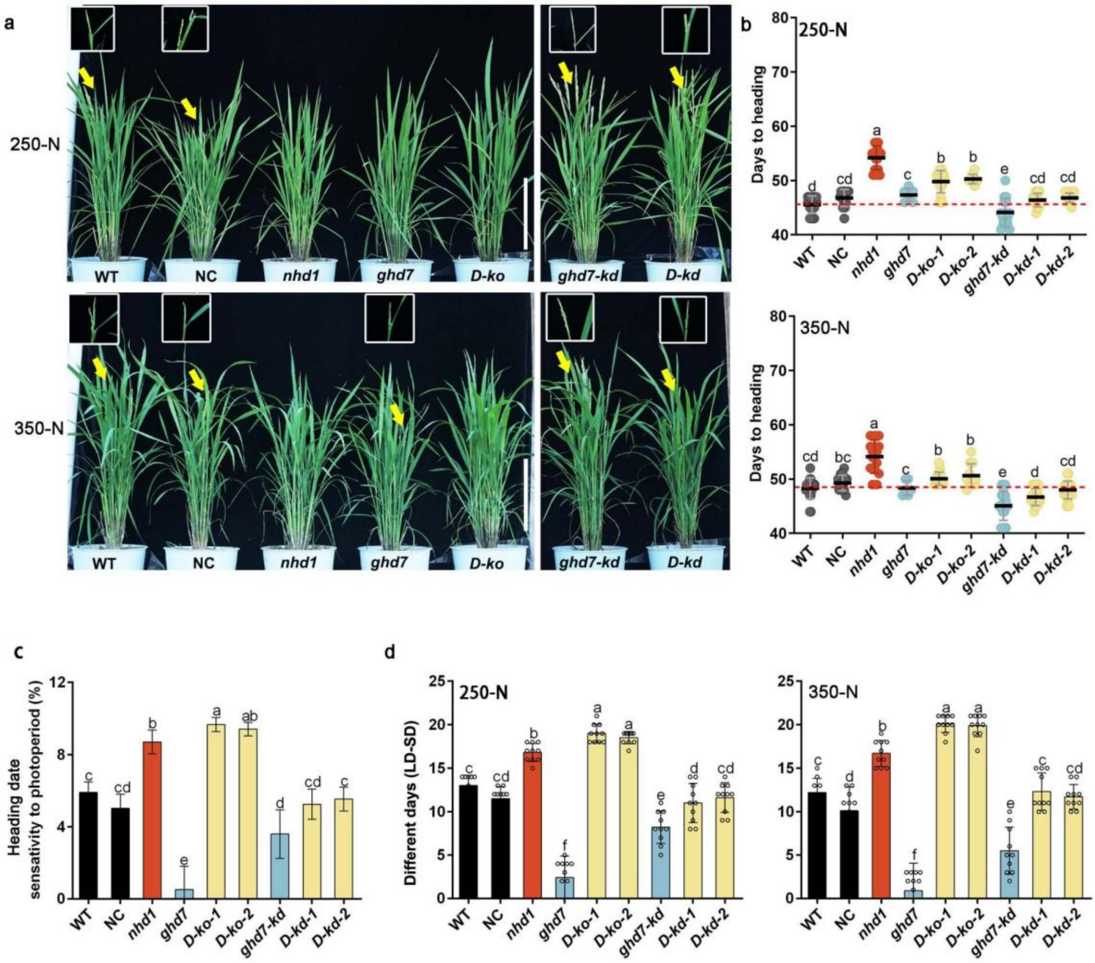

Supplemental figure 4. Sensitivity of flowering time of WT and mutants to photoperiod under different N conditions.

(a-b) Photographs (a) and flowering time (b) of WT, single and double mutants of *ghd7* and *nhd1* plants under SD conditions. the yellow arrow and the amplified frame indicate spikes. Scale bar, 25 cm. Values are means±SD (n≥7). One-way ANOVA was used for the statistical analysis ($p \leqslant 0.05$). (c) Flowering time sensitivity to photoperiod of representative plants. Calculation of Photoperiod sensitivity were descripted in material and method. (d) The difference of flowering time between LD and SD of representative plants growing in 250 kg-N/ha and 350 kg-N/ha conditions. Values are means±SD (n≥7). One-way ANOVA was used for the statistical analysis ($p \leqslant 0.05$）.

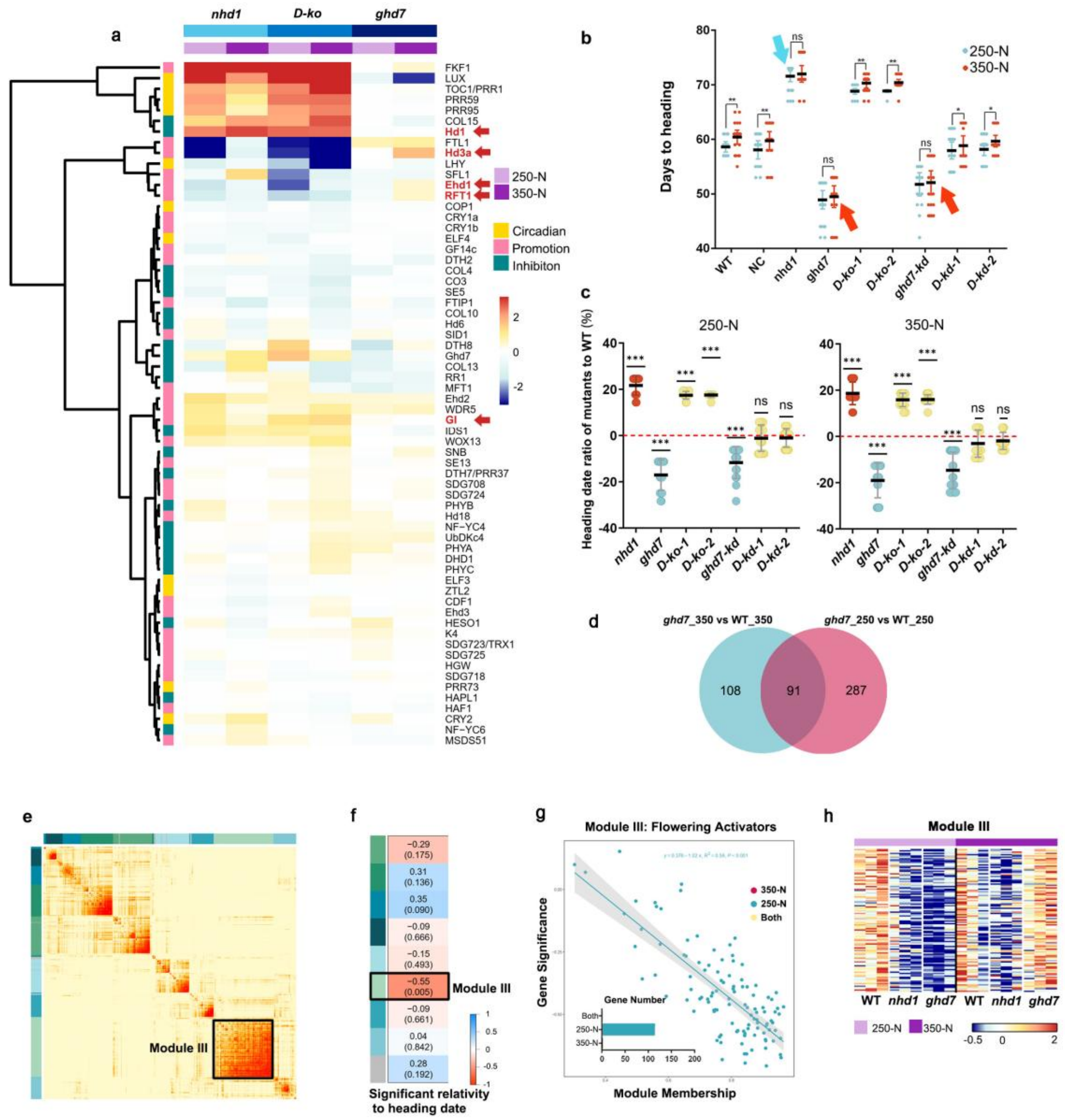

Supplemental figure 5. Ghd7 regulation of flowering time is mainly via Nhd1-dependent pathway.

(a) The heatmap for the up- or down-regulation of known floral genes in single and double mutant of *nhd1* and *ghd7* relative to the WT under 250 kg-N/ha and 350 kg-N/ha treatments. The expression difference value indicates $\log_2$FoldChange of each gene. (b) Days to heading of WT, NC and the single and double mutants of *ghd7* and *nhd1* under 250 kg-N/ha and 350 kg-N/ha conditions. Values are means±SD ($n \geqslant 10$). One-way ANOVA was used for the statistical analysis (ns, $p > 0.05$; *$p \leqslant 0.05$; **$p \leqslant 0.01$; ***$p \leqslant 0.001$). (c) The ratio of flowering time between mutants and WT at different N conditions. Ratio value: [(flowering time of related mutant – flowering time of WT) / flowering time of WT] × 100%. (d) The number of the different expression genes (DEGs) between *ghd7* mutant and WT under 250 kg-N/ha and 350 kg-N/ha conditions. (e) The heatmap plot of the topological overlap-based dissimilarity with hierarchical clustering dendrograms for *ghd7* (see the

WGCNA described in materials and methods). The transformation from yellow to red color indicates low to high topological overlap. (f) The heatmap represents the correlation of the eigengene of each module shown in (c) with heading date, with red colors for positive correlations and blue colors for negative correlations. Texts within the heatmap indicate the correlation coefficients and the corresponding *p*-values (in parentheses). (g) Scatterplots of genes in module III significance for heading date versus module membership in the most significant modules across N levels. (h) Transcription profiling of genes in module III in WT, *nhd1* and *ghd7* under 250 kg-N/ha and 350 kg-N/ha conditions.

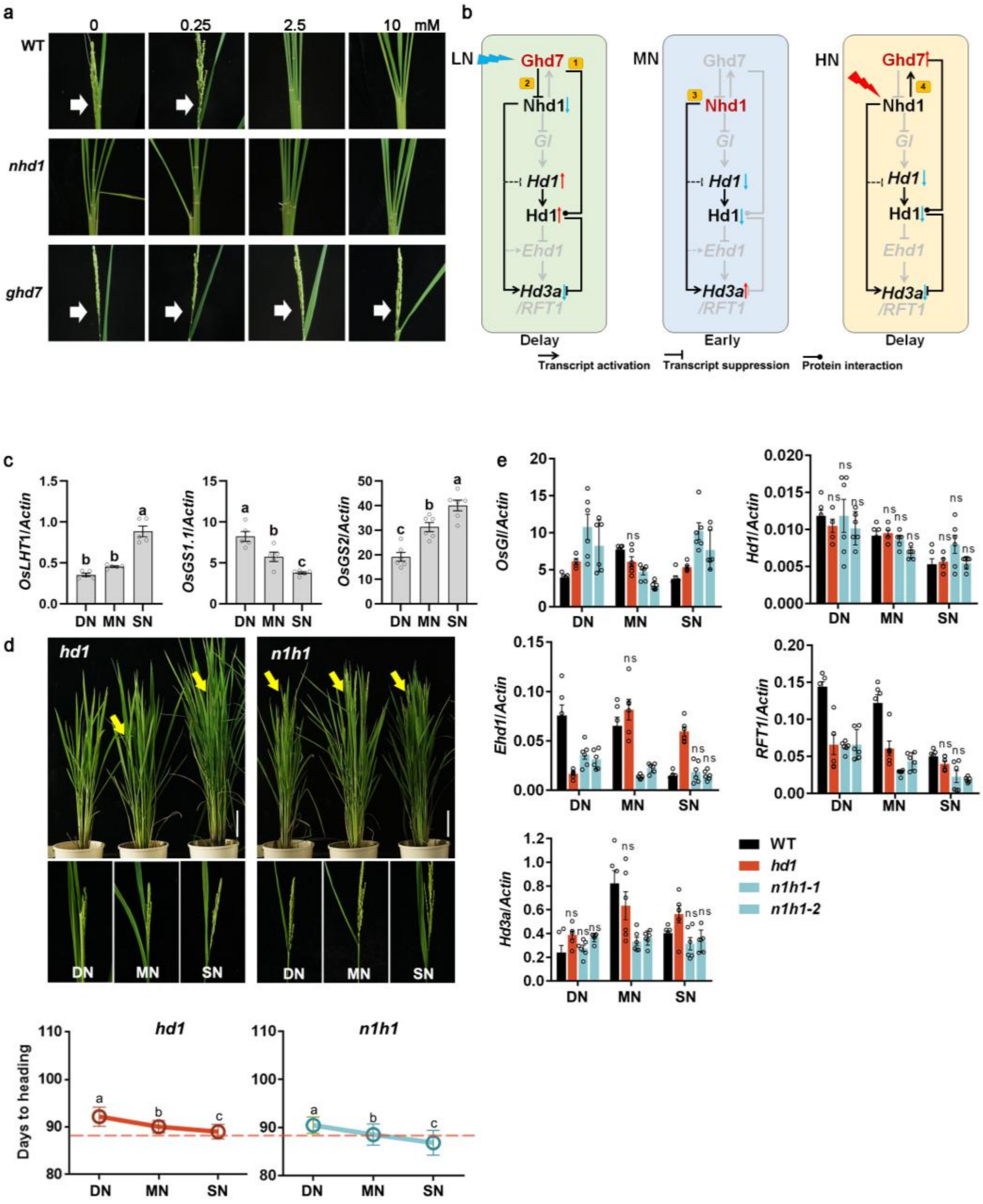

Supplemental figure 6. Nhd1 and Ghd7 are required for the U-shape response of flowering time to the supply of N at broad ranges.

(a) Flowering phenotypes of WT, *nhd1* and *ghd7* plants growing under different N concentration in hydroponic system. Photos were taken in the flowering time of WT under 0.25 mM N condition. (b) Working model of flowering regulation by Nhd1-Ghd7 module under different N conditions. Under LN condition, highly expressed Ghd7 inhibits flowering by downregulating florigens expression through suppressing *Nhd1* transcription (pathway 1) and interacting with Hd1 protein (pathway 2); Under MN condition, the repression of Nhd1 transcription is released as the decrease of *Ghd7* transcription. Consequently, high abundant of *Nhd1* transcription promotes flowering by directly upregulating florigens expression and indirectly downregulating *Hd1* expression (pathway 3); Under HN condition, upregulated Nhd1 significantly activates *Ghd7* transcription, which in turn postpones flowering time by interacting with Hd1 protein to suppress florigens expression (pathway

4). (c) Expression of the N-responsive genes in WT under DN, MN and SN conditions in paddy field. (d) Photographs and days to heading of *hd1* single mutant and *nhd1*/*hd1* (*n1h1*) double mutant under DN, MN and SN conditions in paddy field. (e) Expression of the known downstream flowering genes of Nhd1 and Ghd7 in WT, *hd1* and *n1h1* mutants under different N conditions. Values in (d) are means±SD (n⩾5). Values in (c) and (e) are means ±SE (n⩾3). One-way ANOVA was used for the statistical analysis ($p \leqslant 0.05$). ns indicates no significant difference to WT.

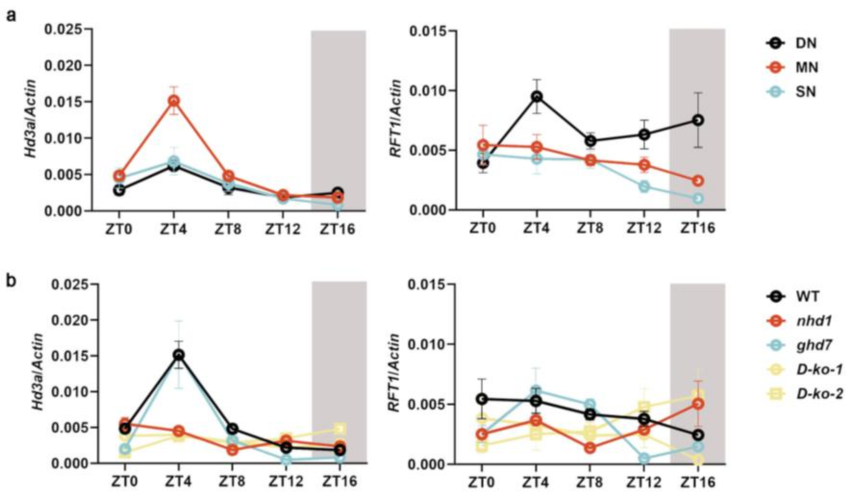

Supplemental figure 7. Diurnal expression of florigens genes in different N conditions and mutants grown in MN condition

(a) Diurnal expression of *Hd3a* and *RFT1* in DN, MN and SN conditions. (b) Diurnal expression of *Hd3a* and *RFT1* in WT, single and double mutants of *nhd1* and *ghd7* in MN condition. Plants were grown in the basal nutrient solution for 1 month and transferred to different N conditions for 15 days. The first leaf of each plant was sampled every 4 hours for gene expression analysis.

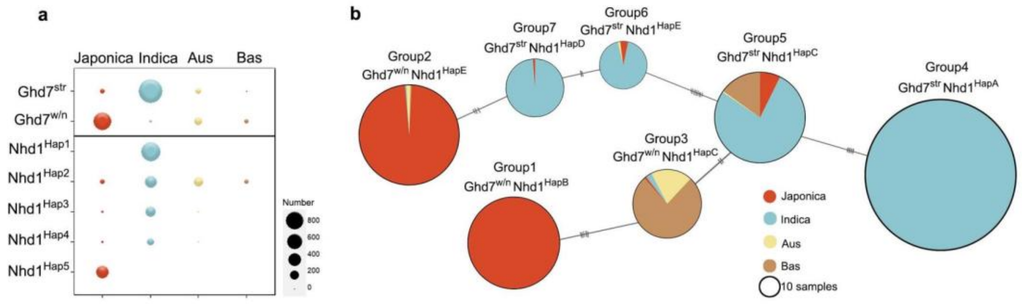

c

Aus/Bas/Ind/Jap — SNP position in *Nhd1* promoter

Groups	Number	Ghd7^Haps	Nhd1^Haps	-2818	-2736	-2479	-2377	-2102	-2101	-1797	-1535	-1432	-1203	-1109	-1091	-914	-809	-536	-370	-131	-47
Group1	0/0/0/190	Ghd7^w/n	Nhd1^HapB	C	C	A	A	T	G	T	A	A	C	A	T	T	G	C	T	C	C
Group2	1/3/1/229	Ghd7^w/n	Nhd1^HapE	G	C	A	G	C	A	C	G	A	C	G	A	G	G	T	C	T	C
Group3	78/20/3/1	Ghd7^w/n	Nhd1^HapC	C	C	A	A	C	G	T	G	A	C	G	T	T	T	C	T	C	T
Group4	0/0/534/0	Ghd7^str	Nhd1^HapA	C	T	G	A	C	G	T	G	G	G	G	T	T	T	C	T	C	T
Group5	35/1/189/17	Ghd7^str	Nhd1^HapC	C	C	A	A	C	G	T	G	A	C	G	T	T	T	C	T	C	T
Group6	0/0/76/1	Ghd7^str	Nhd1^HapE	G	C	A	G	C	A	C	G	A	C	G	A	G	G	T	C	T	C
Group7	0/1/53/3	Ghd7^str	Nhd1^HapD	C	C	A	G	C	A	C	G	A	C	G	A	G	G	C	C	T	C

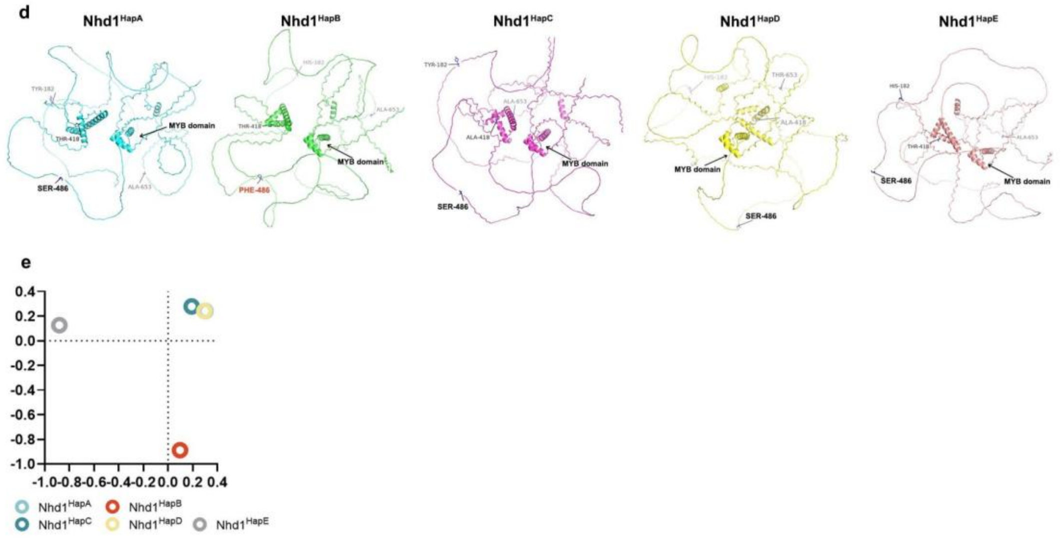

Supplemental figure 8. Natural variation of Nhd1-Ghd7 module controls flowering time.

(a) Distribution of *Ghd7* and *Nhd1* alleles in *Japonica*, *indica*, *Aus* and *Bas*. (b) Classification and proportion of various combinations of different *Ghd7* and *Nhd1* alleles in different rice subspecies. (c) SNPs in *Nhd1*'s promoter of the seven *Ghd7*-*Nhd1* combination groups. (d) Predicted protein structure of different Nhd1 haplotypes using AlphaFold3 (alphafoldserver.com). (e) Correlation analysis of the predicted structures of proteins shown in (d) using DALI protein structure comparison server (http://ekhidna2.biocenter.helsinki.fi/dali/; Holm, 2019). The position of each protein points with the most similar structural neighborhoods near each other (Zilberzwige-Tal et al., 2025).

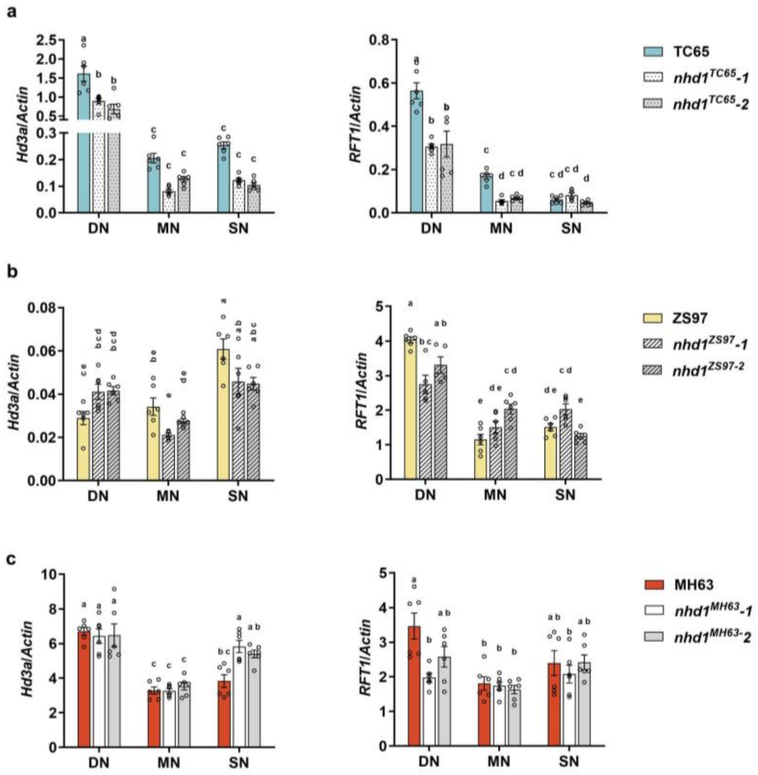

Supplemental figure 9. Expression of florigens genes in *nhd1* mutants generated in different rice cultivars under different N conditions in the field.

(a-c) Expression of *Hd3a* and *RFT1* in *nhd1* mutants generated in TC65 (a), ZS97 (b), and MH63 (c) backgrounds under DN, MN and SN supplies in the field. Values are means±SE (n⩾5). Two-way ANOVA was used for the statistical analysis ($p \leqslant 0.05$).

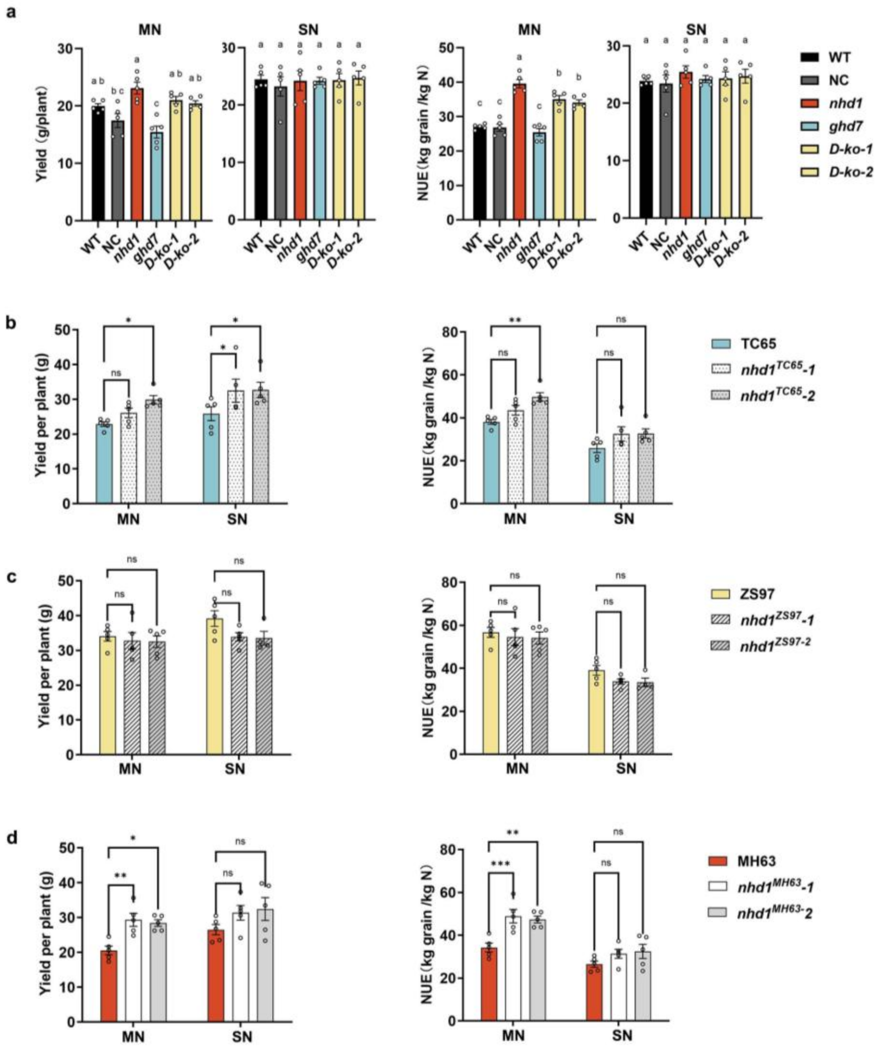

Supplemental figure 10. Yield and nitrogen use efficiency in WT and mutants of different rice cultivars under two N treatments in the field.

(a) Yield and nitrogen use efficiency (NUE) in WT, NC, single and double mutant of *nhd1* and *ghd7* in Nipponbare background under MN and SN supplies in the field. Values are means±SD (n⩾4). one-way ANOVA was used for the statistical analysis ($p \leqslant 0.05$) in different N conditions, respectively. (b-d) Yield and NUE of *nhd1* mutants generated in the TC65 (b), ZS97 (c), and MH63 (d) cultivar backgrounds under MN and SN supplies in the field. MN, 150 kg N/ha; SN, 250 kg N/ha. Values are means±SD (n⩾4). One-way ANOVA was used for the statistical analysis (ns, $p > 0.05$; *$p \leqslant 0.05$; **$p \leqslant 0.01$; ***$p \leqslant 0.001$).
