# Supplementary material for: U‐Shaped Response of Flowering Time to Low and High Nitrogen via a Transcription Feedback Loop in Rice
Source: Adv Sci (Weinh). 2025 Dec 7;13(4):e08498. doi: 10.1002/advs.202508498 (PMC12822397; doi:10.1002/advs.202508498)
Supplement: Supplementary file 2 — Supporting Tables [file ADVS-13-e08498-s001.docx]

| **Supplemental Table 1. Primers sequences used in this study.** | |
| --- | --- |
|  |  |
| **For RT-qPCR** | |
| *OsActin-F* | CAACACCCCTGCTATGTACG |
| *OsActin-R* | CATCACCAGAGTCCAACACAA |
| *OseEF-1a-F* | TTTCACTCTTGGTGTGAAGCAGAT |
| *OseEF-1a-R* | GACTTCCTTCACGATTTCATCGTAA |
| *Nhd1-F* | GGGTCGTCTGGCTTTTGAT |
| *Nhd1-R* | CGGTACCCTGTTCTCCTTC |
| *Ghd7-D-F* | AGGTGCTACGAGAAGCAAATCC |
| *Ghd7-D-R* | GGGCCTCATCTCGGCATAG |
| *OsGI-F* | GTTTCCCGTTCATGTCCT |
| *OsGI-R* | TCCGCTTCACTATGTTGG |
| *OsHd1-F* | TCAGCAACAGCATATCTTTCTCATCA |
| *OsHd1-R* | TCTGGAATTTGGCATATCTATCACC |
| *OsHd3a-F* | GCTCACTATCATCATCCAGCATG |
| *OsHd3a-R* | CCTTGCTCAGCTATTTAATTGCATAA |
| *OsRFT1-F* | TGACCTAGATTCAAAGTCTAATCCTT |
| *OsRFT1-R* | TGCCGGCCATGTCAAATTAATAAC |
| *Ehd1-F* | TACAATGGCGAGATCACGGA |
| *Ehd1-R* | AACTGCCTTGTCTTCTCCGA |
| *OsLHT1-F* | GGACTCCGGCAGATCATCA |
| *OsLHT1-R* | CTGGTTTCATCATGTGTGCCTA |
| *OsGS1.1-F* | TGTGGTATCGGTGCTGACAAG |
| *OsGS1.1-R* | AACTCCCACTGTCCTGGCAT |
| *OsGS2-F* | AGTGTCGGTATTGAAGCTGGAG |
| *OsGS2-R* | ACGCATACTCTTGGTGCTGTAG |
| **For Yeast One-Hybrid assay** | |
| *AD-Nhd1-F* | GAATTCATGGAGATTAATTCCTCTGG |
| *AD-Nhd1-R* | CCTAGGTCATGTCGATGCTTCGCTCT |
| *AD-Ghd7-F* | GAATTCATGTCGATGGGACCAGCAGC |
| *AD-Ghd7-R* | CCTAGGCTATCTGAACCATTGTCCAA |
| *pNhd1-F* | TGGTGGAGTTCAGTTTGACCATTTG |
| *pNhd1-R* | TCCCAATTCAAGAAAAAGAAGAAAT |
| *pGhd7-F* | ACCGCACGCAAAAATCTTTATTGTAGT |
| *pGhd7-R* | CTACAATAAAGATTTTTGCGTGCGGTC |
| **For ChIP-qPCR** | |
| *ProGhd7-F* | TCCGCGCAGAAAAATTATTTTCGAC |
| *ProGhd7-R* | CCCTCCATTTCAGTTATAAGACGTT |
| *ProNhd1-F* | TGCGTTGACATGATGTTAAAGTTAC |
| *ProNhd1-R* | CTTTCTTAGTGGTGGCAACAACCTA |
| **For EMSA assay** | |
| *Motif-NBS-F-Bio* | 5'Biotin+TTTTCGACCGCACGCAAAAATCTTTATTGTAGTAGTGT |
| *Motif-NBS-F* | TTTTCGACCGCACGCAAAAATCTTTATTGTAGTAGTGT |
| *Motif-NBS-R* | ACACTACTACAATAAAGATTTTTGCGTGCGGTCGAAAA |
| *Motif-EEL-F-Bio* | 5'Biotin+CAAATGGTTGAGATATTTAGTTTCGCGCC |
| *Motif-EEL-F* | CAAATGGTTGAGATATTTAGTTTCGCGCC |
| *Motif-EEL-R* | GGCGCGAAACTAAATATCTCAACCATTTG |
| *EMSA-Ghd7-F* | GAGCTCATGTCGATGGGACCAGCAGCCGGAG |
| *EMSA-Ghd7-R* | AAGCTTCTATCTGAACCATTGTCCAAGCTCA |
| *EMSA-Nhd1-F* | GCTTCCATGGAGATTAATTCCTCTGG |
| *EMSA-Nhd1-R* | GAGCTCTGTCGATGCTTCGCTCT |
| **For transient transactivation assay** | |
| *Nhd1-CDS-F* | GGGGACAAGTTTGTACAAAAAAGCAGGCTTAATGGAGATTAATTCCTCTGGTGAGG |
| *Nhd1-CDS-F* | GGGGACCACTTTGTACAAGAAAGCTGGGTATCATGTCGATGCTTCGCTCTCAAGA |
| *Ghd7-CDS-F* | GAGCTCATGTCGATGGGACCAGCAGC |
| *Ghd7-CDS-R* | GGATCC AATTATCTATCTGAACCATT |
| *pGhd7-F-LUC* | AAGCTTGGGTGATATAGCGGCAGCGC |
| *pGhd7-R-LUC* | GGATCCGAACGGATAAATCAAACTCG |
| *pNhd1-F-LUC* | AAGCTTAGATCTTGGTTACCTTGTTCGTGCA |
| *pNhd1-R-LUC* | GGATCCTCCCAATTCAAGAAAAAGAAGAAAT |

**Supplemental Table 2. Days to heading of rice varieties in group 1, group 2, group 4, group 5 and group 6 under MN and HN conditions.**

| **Groups** | **Days to heading** | |
| --- | --- | --- |
| **Group 1 (n=18)** | **MN** | **SN** |
| Nipponbare_japonica | 116 | 126 |
| LAC_23_japonica | 93 | 103 |
| CUNSANLI_japonica | 105 | 116 |
| GERDEH_japonica | 103 | 105 |
| WEIGUO_japonica | 103 | 105 |
| HEIGENG_2_japonica | 103 | 105 |
| XIANGQING_japonica | 108 | 110 |
| GENG_87-304_japonica | 109 | 111 |
| GUIHUAHUANG_japonica | 103 | 104 |
| HUANGKEZAONIAN_japonica | 103 | 104 |
| LAOHUZHONG_japonica | 108 | 107 |
| FEIDONGTANGDAO_japonica | 103 | 102 |
| IGUAPE_CATETO_japonica | 108 | 105 |
| MUXIQIU_japonica | 103 | 99 |
| TODOROKIWASE_japonica | 108 | 103 |
| BALILLA_japonica | 111 | 103 |
| CHIKENUO_japonica | 123 | 108 |
| ZHONGHUA_11_japonica | 111 | 96 |
| **Group 2 (n=9)** | **MN** | **SN** |
| R6_HAOGANG_japonica | 106 | 126 |
| HONGMISANDAN_japonica | 96 | 108 |
| HANDAO_8_japonica | 93 | 103 |
| HAOBUKA_japonica | 98 | 103 |
| CUNGUNUO_japonica | 108 | 111 |
| SANBANGQISHILUO_japonica | 111 | 114 |
| HONGKEZHENUO_japonica | 116 | 118 |
| DANDONGLUDAO_japonica | 102 | 103 |
| LIJIANGXINTUANHEIGU__japonica | 111 | 124 |
| **Goup 4 (n=26)** | **MN** | **SN** |
| LUCAIHAO_indica | 101 | 124 |
| ZAOXIAN_240_indica | 103 | 111 |
| ZHENXIAN_232_indica | 108 | 116 |
| XIAOHONGGU_indica | 116 | 124 |
| ZAOSHUXIANGHEI_indica | 103 | 110 |
| XIANGWANXIAN_1_indica | 103 | 110 |
| AIJIAONANTE_indica | 103 | 108 |
| MAMAGU_indica | 99 | 103 |
| HEIDU_4_indica | 101 | 103 |
| ZHONG_413_indica | 103 | 105 |
| IAC_1246_japonica | 126 | 128 |
| YOUNIAN_indica | 103 | 104 |
| SANLICUN_indica | 116 | 117 |
| GUICHAO_2_indica | 108 | 108 |
| ZHUZHEN_B_indica | 108 | 108 |
| SHANHUANGZHAN_2__indica | 111 | 110 |
| NANJING_11_indica | 103 | 102 |
| QITOUGU_indica | 116 | 113 |
| SANKECUN_indica | 111 | 108 |
| XIANGAI_B_indica | 111 | 108 |
| TEQING_indica | 116 | 108 |
| XIBAINIAN_indica | 126 | 116 |
| JINZHINUO_indica | 128 | 115 |
| XIANGAIZAO_10_indica | 130 | 116 |
| JINXIBAI_indica | 116 | 116 |
| WUZUIHONGGU_indica | 124 | 116 |
| **Goup 5 (n=9)** | **MN** | **SN** |
| C129_AIHECHI_indica | 90 | 124 |
| R93_RIKUTO_NORIN_21_indica | 103 | 111 |
| C197_MAWEINIAN_indica | 109 | 115 |
| R86_DUOCHE_indica | 108 | 112 |
| R81_IR_30358-084-1-1_indica | 108 | 108 |
| C015_DULAR_aus | 116 | 116 |
| C182_HANGXIANLIANGCHUN_indica | 111 | 110 |
| R5_C_22_indica | 111 | 108 |
| C183_LEIHUOZHAN_indica | 111 | 103 |
| **Goup 6 (n=7)** | **MN** | **SN** |
| BALA_indica | 116 | 124 |
| HEGANXIANNIAN_indica | 103 | 107 |
| IAC_5100_indica | 108 | 111 |
| DALAI_AMAN_indica | 109 | 110 |
| DANGYU_5_indica | 116 | 115 |
| T_2095_indica | 108 | 108 |
| BAYUENUO_indica | 103 | 106 |

**Supplemental Table 3. Z scores of alignments between each predicted protein of different Nhd1 haplotypes**

|  | Nhd1^HapA^ | Nhd1^HapB^ | Nhd1^HapC^ | Nhd1^HapD^ |
| --- | --- | --- | --- | --- |
| Nhd1^HapB^ | 7.4 |  |  |  |
| Nhd1^HapC^ | 7.4 | 7.4 |  |  |
| Nhd1^HapD^ | 15.2 | 7.4 | 13.6 |  |
| Nhd1^HapE^ | 8.5 | 6.0 | 9.7 | 8.6 |
